# Compliance and Retention With the Experience Sampling Method Over the Continuum of Severe Mental Disorders: Meta-Analysis and Recommendations

**DOI:** 10.2196/14475

**Published:** 2019-12-06

**Authors:** Hugo Vachon, Wolfgang Viechtbauer, Aki Rintala, Inez Myin-Germeys

**Affiliations:** 1 Center for Contextual Psychiatry, Department of Neurosciences KU Leuven Leuven Belgium; 2 School for Mental Health and Neuroscience, Department of Psychiatry and Neuropsychology Maastricht University Maastricht Netherlands

**Keywords:** experience sampling, ecological momentary assessment, compliance, retention, severe mental disorders

## Abstract

**Background:**

Despite the growing interest in the experience sampling method (ESM) as a data collection tool for mental health research, the absence of methodological guidelines related to its use has resulted in a large heterogeneity of designs. Concomitantly, the potential effects of the design on the response behavior of the participants remain largely unknown.

**Objective:**

The objective of this meta-analysis was to investigate the associations between various sample and design characteristics and the compliance and retention rates of studies using ESM in mental health research.

**Methods:**

ESM studies investigating major depressive disorder, bipolar disorder, and psychotic disorder were considered for inclusion. Besides the compliance and retention rates, a number of sample and design characteristics of the selected studies were collected to assess their potential relationships with the compliance and retention rates. Multilevel random/mixed effects models were used for the analyses.

**Results:**

Compliance and retention rates were lower for studies with a higher proportion of male participants (*P*<.001) and individuals with a psychotic disorder (*P*<.001). Compliance was positively associated with the use of a fixed sampling scheme (*P*=.02), higher incentives (*P*=.03), higher time intervals between successive evaluations (*P*=.02), and fewer evaluations per day (*P*=.008), while no significant associations were observed with regard to the mean age of the sample, the study duration, or other design characteristics.

**Conclusions:**

The findings demonstrate that ESM studies can be carried out in mental health research, but the quality of the data collection depends upon a number of factors related to the design of ESM studies and the samples under study that need to be considered when designing such protocols.

## Introduction

### Background

The experience sampling method (ESM) [1] or ecological momentary assessment (EMA) [2] can be used interchangeably to refer to an assessment method that involves the collection of repeated and momentary self-evaluations in the context of an individual’s daily life. Compared with conventional clinical tools that are typically administered once and in a lab/clinical setting, this methodology improves ecological validity, limits potential artifacts because of retrospective recall [3‑6], can capture the within-person fluctuations of psychological states and behaviors [7‑9], and allows for a more fine-grained examination of contextual factors [10‑12]. As such, ESM is of particular interest in clinical psychology where patients are affected by memory problems [3,4], unstable affective states [5,6], and by a heightened sensitivity to contextual factors [17‑19]. ESM has, therefore, been extensively used in this field of research over the past 30 years [7,8], particularly in populations with depressive disorders [7,9] and psychosis [8,10].

Although ESM presents several advantages over conventional clinical assessment tools, the very nature of this method, requiring multiple self-evaluations over time in daily life, also introduces some challenges. One major challenge is to achieve high compliance and retention rates. The compliance rate can be defined as the ratio of the number of self-evaluations that participants actually completed over the theoretical maximum number of self-evaluations allowed by the protocol (0%‑100% when expressed as a percentage), whereas the retention rate refers to the proportion (or percentage) of participants included in the final analyses (eg, a subject withdrawing their participation from a study, for example, because the data collection procedure is experienced to be too burdensome, would be excluded). These two rates are often inherently linked in ESM research, as participants providing an insufficient number of responses are conventionally excluded from the analyses [11], which in turn influences the retention rate.

In the framework of ESM, compliance and retention rates are often reported to describe the quantity of data collected and to provide an indication of the quality of the data collection procedures. ESM studies are naturalistic investigations, inevitably leading to missing data. When people are engaging in certain sport, leisure, or work activities, driving in their car, or taking a nap, they will not be able to fill out the ESM questionnaire (either because they do not hear the notification of the data collection device or because responding would be inconvenient, unsafe, or inappropriate to do in a given situation). Compliance rates close to 100% are therefore unlikely. Yet, ideally, one wants to reach the highest compliance possible, as this alleviates concerns about selective reporting at moments that are most convenient for the study participants (which could lead to bias). At the same time, we also need a sufficient number of data points to investigate, for example, variability over time, and to estimate stable associations between variables measured using this method. It is, therefore, important to identify how characteristics of both the ESM design and the samples under investigation influence compliance and retention. Using this information, we might be able to identify designs that are more acceptable to a given group of study participants.

To our knowledge, whether design and sample characteristics influence retention has not been the focus of prior research, but several studies have examined this question with respect to compliance. Compliance tends to decrease over the duration of the ESM follow-up [12], during the early mornings [26‑28], the evenings [13], in the middle of the week [14], outside home [15], when questionnaires encompass more items [16], when successive self-evaluations are separated by longer periods [15], and in the absence of incentives [16]. In addition, even if not directly targeting compliance, Stone et al [17] found that the number of daily self-evaluations correlated significantly with an increased perception of burdensomeness, which may indirectly impact compliance. In other words, compliance may be tightly related to methodological aspects that researchers could adjust to increase the amount of data collected and to enhance the acceptability of ESM for study participants.

The ESM literature displays a rather heterogeneous methodological landscape. Designs vary from 2 [18] to 50 evaluations per day [19], occurring at fixed [20], semirandom [21], or random time intervals [22], for 1 [23] to 150 days [24], using paper-and-pencil [25] or electronic devices [26], Likert scales [27], or visual analogue scales [28], and with questionnaires varying in length from 2 [29] to 100 items [30]. In addition, Janssens et al [31] argued that the methodological choices in designing ESM research are often guided more by practical considerations (contextual constraints, statistical requirements, and replication of existing protocols) rather than based on theory or evidence. Thus, whereas these decisions may have considerable influence on the quality of the data collection, there is currently a lack of empirical evidence to guide researchers when designing their ESM protocols.

The compliance rate in ESM studies may also be influenced by the individual characteristics of the study samples. Indeed, compliance appears to drop in relation to the ratio of male participants [14,32], in substance users [14], alcohol users [15], and in younger samples [16], but also in individuals with higher levels of negative affect [15], or in those with a psychotic disorder [32], putting clinical samples at particular risk for exhibiting low compliance levels.

Therefore, both design- and participant-related factors may influence compliance. Fortunately, compliance is typically reported within the ESM literature, making this information highly accessible for a meta-analysis over a large sample of studies. To date, two studies have addressed this question through a meta-analysis. Morren et al [16] demonstrated the effect of several design- (ie, length of ESM questionnaires, use of an alarm, and use of an incentive) and participant-related (ie, age and gender of the sample) characteristics on the compliance rate in ESM studies. Conversely, Jones et al [33] did not observe any effect of design characteristics (ie, frequency of evaluations, duration of the study, and device) or of clinical status (ie, substance use) on compliance. However, these reviews focused on patients with chronic pain and substance users, respectively, which limits the comparability of their findings and, importantly, the generalizability to other clinical samples. Finally, the potential influence of design and sample characteristics on the retention rate in ESM research remains unexplored.

### Objective

This meta-analysis, therefore, aims to fill this gap and examines compliance and retention in ESM studies focusing on severe mental disorders, investigating the effect of a large set of design‑ and participant‑related factors with the aim to provide, if achievable, empirically-based guidelines that could support researchers’ choices in designing ESM protocols.

## Methods

### Protocol Registration

This study was based on the PRISMA-P (Preferred Reporting Items for Systematic Review and Meta-Analysis Protocols) guidelines [34]. The protocol has been registered in the International Prospective Register of Systematic Reviews database (PROSPERO 2017: CRD42017060322) and is described in more detail elsewhere [35].

### Data Sources and Literature Search

A systematic literature search was performed until February 2017 without publication time limit in PubMed and Web of Science (ie, Web of Science Core Collection). The search strategy was designed to include relevant terms for identifying studies using momentary assessment methods (eg, “experience sampling method” and “ecological momentary assessment”) and terms related to the clinical diagnosis of the participants under study (eg, “psychotic disorder”, “major depressive disorder”, and “bipolar disorder”). The search strategy used either Medical Subject Heading or keyword headings. A concept plan was built with the identified keywords and descriptors to run the search (see Multimedia Appendix 1).

### Inclusion and Exclusion Criteria

Studies using ESM/EMA designs in adults with a psychotic disorder, major depressive disorder, bipolar disorder, or at high risk for these disorders, and samples of the general population including individuals with or at high risk for these disorders have been included in this review to cover a broader range of the continuum of mood and psychotic disorders. Observational and randomized controlled studies have been included. Case studies, case reports, protocols, descriptions of study designs, systematic reviews, and studies published in a language other than English have not been considered. When available within the included studies, data from nonpsychopathological/healthy control groups have also been considered to serve as a reference group. Studies with only a single daily assessment have been excluded as this form of time sampling is qualitatively distinct from the repeated momentary assessments within a day that defines ESM research. To determine the eligibility of the original studies, two researchers (HV and AR) independently conducted the screening of the studies in the title/abstract and full-text phases based on the inclusion and exclusion criteria. Screening results were compared with identify any discrepancies. In case of a disagreement, a third researcher (IM-G) was consulted and the discrepancy was resolved through group consensus.

### Data Extraction

When available, data were extracted for the following items: (1) general study characteristics (ie, authors, title, year, and study design); (2) sample characteristics (ie, number of participants included in the study/analysis, mean age, gender composition, clinical status, ethnicity, educational status, employment status, marital status, cohabiting status, and medication use); (3) design characteristics (ie, number of momentary assessments per day, number of assessment days, number of assessment periods as continuous or intermittent assessment, delay between assessment periods, sampling method [fixed, semirandom, or random sampling], time intervals between the assessments within a day, time intervals between the first and the last assessment within a day, time of the start and the end of the assessments within a day, number of items in the questionnaire, approximate mean duration of the questionnaire, type of scales used in the questionnaire, type of method used to perform the assessment, type of incentive, and amount of the incentive); and (4) the compliance rate (proportion of self-evaluations completed by the participants compared with the theoretical maximal number of self-evaluations allowed by the design) and the retention rate (proportion of individuals included in the final analysis out of the number of individuals included at baseline). For studies that included multiple groups (eg, a psychotic disorder group and a healthy control group), sample/design characteristics and the compliance and retention rates were coded at the group level. Studies that fulfilled the inclusion criteria were examined for overlapping samples (Multimedia Appendix 1). When needed, the corresponding authors of the original studies were contacted for further information. Data from the included studies have been extracted and stored in a customized spreadsheet structured according to the items mentioned above, which is provided as part of the Multimedia Appendix 1.

### Risk of Bias

According to the PRISMA guidelines, risk of bias should be assessed for each study (eg, lack of blinding, lack of randomization). However, the current review did not investigate randomized controlled trials and neither compliance nor retention rates were primary outcomes within the sample of studies included in the meta-analysis. Additionally, there is to date no standardized risk of bias assessment guideline for ambulatory studies. The evaluation of the risk of bias was therefore not performed (although we did examine the data for potential publication bias; see further below).

### Statistical Analysis

**Figure 1:**
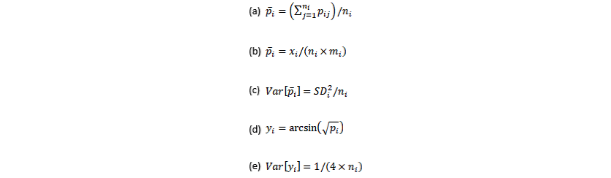
Equations.

For compliance, there is, in principle, a proportion of completed self-evaluations per participant (eg, 0.80 for the first subject, 0.65 for the second subject, and so on), but this information is never reported. Instead, we analyzed the mean proportions (equation [a], Figure 1), where *p_ij_* denotes the proportion of completed evaluations for the *j*th participant in the *i*th group and *n_i_* the group size). We expected either *p_i_* to be reported directly (either in terms of a proportion or percentage) or the total number of self-evaluations collected, which is easily converted to *p_i_* (equation [b], Figure 1), where *x_i_* denotes the total number of self-evaluations collected and *m_i_* the theoretical maximal number of self-evaluations per subject as allowed by the design). The sampling variance of *p_i_* was computed following equation (c) (equation [c], Figure 1), where *SD_i_* is the SD of the compliance rates of the *n_i_* subjects in the *i*th group. As *SD_i_* was not available for approximately half of the groups, we imputed missing *SD_i_* values based on the expected quadratic relationship between *p_i_* and *SD_i_* (ie, *SD_i_* must be 0 for *p_i_* equal to 0 and 1 and will peak around *p_i_*=0.5). For this, we first meta-analyzed the available log-transformed *SD_i_* values [36] using a mixed effects meta-regression model with *p_i_* and *p_i_^²^* as predictors and then imputed missing *SD_i_* values based on the fitted values from this model (Multimedia Appendix 1).

For the analysis of the retention rates, the reported/calculated proportions (of individuals included in the final analysis compared with the number of individuals included at baseline) were first transformed using the (variance-stabilizing) arcsine transformation before the analysis (equation [d], Figure 1), where *p_i_* is the proportion of individuals in the *i*th group that were retained for the final analysis [37]. This allowed the inclusion of groups with perfect (ie, 100%) retention rates (which occurred in about a quarter of the groups) without the need to make use of continuity corrections. The sampling variance of the transformed proportions was computed following equation (e), Figure 1.

As a study may include multiple groups, we used a multilevel random/mixed effects model [38] with random effects for studies and groups within studies for the analysis of both outcomes. The overall mean compliance and retention rates, averaged over groups and studies, were estimated using intercept-only models. The influence of the various sample and design characteristics on the outcomes was examined by including such characteristics as predictors in the models. Group type (6 levels: healthy control, general population, high risk, major depressive disorder, bipolar disorder, and psychotic disorder), ESM sampling scheme (3 levels: fixed, semirandom, and random), data collection method (7 levels: paper-and-pencil, personal digital assistant [PDA], Web-based, call, SMS, voicemail, and mixed), and scale type (3 levels: Likert scale, visual analogue scale, and mixed) were included as factors in the models. All other design characteristics (eg, duration of the ESM follow-up and frequency of the daily evaluations) and sample characteristics (eg, mean age of the sample) were included as continuous predictors in the models. Each of the design and sample characteristics was examined separately. All models were fitted using restricted maximum likelihood estimation, using the R metafor package [39] for the analyses. For the intercept-only models, we report the estimated mean rates (as percentages and after back transformation of the mean arcsine rate for retention) with corresponding 95% CIs. For the meta-regression models, we report the model coefficients, corresponding standard errors, tests and 95% CIs of the individual coefficients, and, for models containing factors, the Q_M_ test of the factor as a whole. For each meta-regression model, we also report pseudo-R^2^-type values [40] for the between-study and between-group variance accounted for by the moderator included in the model.

Heterogeneity was assessed using the Q-test [41] and based on the estimates of the between-study and between-group heterogeneity variance components (with 95% profile likelihood confidence intervals). The presence of outliers or influential studies was determined based on using Cook distance [42] and by examining the distribution of the standardized residuals and the predicted random effects at the group and study levels. Funnel plots and meta-regression models using sample size as predictor were used to examine the data for funnel plot asymmetry.

## Results

**Figure 2:**
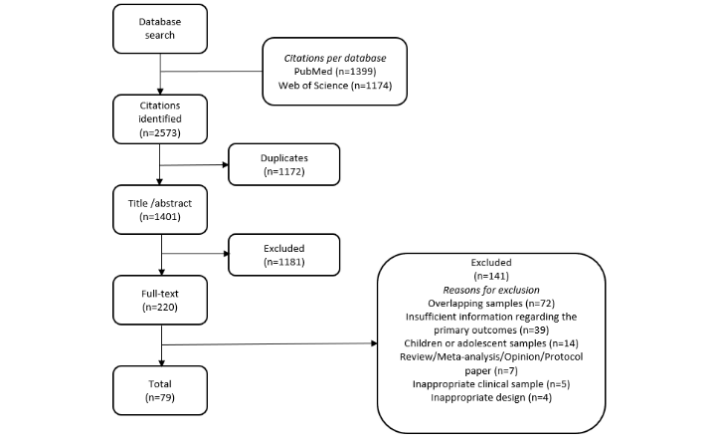
Flow chart of study inclusion protocol.

After screening based on title and abstract, a total of 220 studies were considered for inclusion (Figure 2). Of these, 141 were excluded for reasons as outlined in Figure 2. Finally, 79 studies fulfilled all inclusion criteria (Multimedia Appendix 1). Table 1 shows the characteristics of the studies included in the meta-analysis.

**Table 1:** Descriptive statistics of the sample of studies (N=79).

Characteristics			Study level, n (%)	Group level, n (%)
**General characteristics**				
	**Year of publication**			
		<2000	4 (5)	N/A^a^
		2000‑2004	4 (5)	N/A
		2005-2009	10 (13)	N/A
		2010-2014	41 (52)	N/A
		≥2015	20 (25)	N/A
	**Sample size**			
		0-49	24 (30)	80 (61)
		50-99	26 (33)	32 (24)
		100-149	14 (18)	9 (7)
		150-199	6 (8)	4 (3)
		≥200	9 (11)	7 (5)
	**Number of groups per study**			
		1	42 (53)	N/A
		2	26 (33)	N/A
		3	7 (9)	N/A
		4	3 (4)	N/A
		5	1 (1)	N/A
**Sample characteristics**				
	**Age (years)**			
		18-29	29 (37)	39 (30)
		30-39	23 (29)	45 (34)
		40-49	15 (19)	27 (21)
		≥50	3 (4)	5 (4)
		Unavailable	9 (11)	16 (12)
	**Gender (% female)**			
		<25	4 (5)	11 (8)
		25-49	18 (23)	28 (21)
		50-74	34 (43)	57 (43)
		≥75	17 (22)	26 (20)
		Unavailable	6 (8)	10 (8)
	**Clinical status**			
		Healthy controls	N/A	33 (25)
		General population	N/A	19 (14)
		High risk for a severe mental disorder	N/A	10 (8)
		Major depressive disorder	N/A	30 (23)
		Bipolar disorder	N/A	9 (7)
		Psychotic disorder	N/A	31 (24)
**Design characteristics**				
	**Number of days**			
		1-5	12 (15)	20 (15)
		6-10	54 (68)	94 (71)
		>10	13 (17)	18 (14)
	**Number of evaluations/day**			
		2-3	11 (14)	17 (13)
		4-5	23 (29)	33 (25)
		6-7	6 (8)	11 (8)
		8-9	9 (11)	13 (10)
		10	27 (34)	54 (41)
		>10	2 (3)	3 (2)
		Unavailable	1 (1)	1 (1)
	**Sampling scheme**			
		Fixed	14 (18)	23 (17)
		Semirandom	32 (41)	55 (42)
		Random	31 (39)	51 (39)
		Unavailable	2 (3)	3 (2)
	**Number of items**			
		<20	36 (46)	58 (44)
		20-39	24 (30)	36 (27)
		40-59	8 (10)	15 (11)
		≥60	1 (1)	4 (3)
		Unavailable	10 (13)	19 (14)
	**Scale type**			
		Likert scale	46 (58)	80 (61)
		Visual analogue scale	8 (10)	11 (8)
		Mixed	23 (29)	38 (29)
		Unavailable	2 (3)	3 (2)
	**Data collection method**			
		Paper	27 (34)	50 (38)
		Personal digital assistant	42 (53)	66 (50)
		Other	11 (14)	16 (12)
	**Compliance rate (%)**			
		50-59	3 (4)	7 (5)
		60-69	8 (10)	12 (9)
		70-79	24 (30)	39 (30)
		80-89	21 (27)	35 (27)
		≥90	9 (11)	16 (12)
		Unavailable	14 (18)	23 (17)
	**Retention rate (%)**			
		50-59	1 (1)	2 (1)
		60-69	4 (5)	6 (4)
		70-79	10 (13)	13 (10)
		80-89	11 (14)	19 (14)
		≥90	46 (58)	76 (58)
		Unavailable	7 (9)	16 (12)

^a^N/A: not applicable.

### Descriptive Information

The final sample of studies comprised 8013 individuals from 132 different groups (with 1‑5 groups per study). The mean age of the individuals was 31.7 years (SD 10.3, range of the mean age of the groups=18‑71.9), and 62.79% (5032/8013) of the participants were female (SD 23.1, range of the percentage of females in the groups=6.7%‑100%). Overall, 1282 (1282/8013, 16.00%) were individuals without a diagnosis of psychiatric illness, 3456 (3456/8013, 43.13%) were recruited from the general population, 1423 (1432/8013, 17.76%) were diagnosed with a psychotic disorder, 1326 (1326/8013, 16.55%) were diagnosed with major depressive disorder, 266 (266/8013, 3.32%) were diagnosed with bipolar disorder, and 260 (260/8013, 3.24%) were diagnosed with a high risk for one of the mental disorders under study.

From a design perspective, ESM studies included in the meta-analysis involved a mean of 6.9 evaluations per day (SD 3.0, range 2‑14) for 11.2 days (SD 19.0, range 1‑150) for a total mean number of 60.2 evaluations per study (SD 45.0, range 8‑300). Successive evaluations within a day were separated by an average of 131.2 min (SD 92.8, range 45‑720) and participants were required to fill in evaluations during a mean total time window of 13.5 h per day (SD 2.2, range 3-17). The sampling scheme was random in 39.2%, semirandom in 40.5%, and fixed in 17.7% of the studies. On average, 22.5 items per questionnaire were collected by the ESM studies (SD 18.6, range 2‑135). As compensation, the mean value of the incentives for the completion of the ESM studies was €63.6 (SD 69, range 0‑350).

Other variables such as ethnicity, education level, marital status, or other design parameters (eg, continuous or intermittent assessment, approximate mean duration of the questionnaire, type of incentive, and strategies taken by the researchers to maintain/increase retention and compliance) may be relevant for the association with compliance and retention, but were reported inconsistently or by too few studies to be taken into account.

### Meta-Analyses of the Compliance and Retention Rates

Mean compliance was reported in 65 (65/79, 82%) of the studies, whereas retention rate was reported in 73 (73/79, 92%) of the studies, and 58 (58/79, 73%) of the studies reported both compliance and retention rates. All studies included in the analysis reported at least one of these main outcomes. At the group level, compliance rates were available for 109 (109/132, 82.6%), and retention rates were available for 116 (109/132, 87.9%) of the groups (see Multimedia Appendix 1 for forest plots). On the basis of the multilevel models, the estimated average compliance was 78.7% (95% CI 76.2 to 81.2), and the estimated average retention was 93.1% (95% CI 90.8 to 95.1). However, 2 studies with very low compliance rates [43,44] and 3 studies with very low retention rates [44-46] were found to be overly influential based on their Cook distances (larger than the median Cook distance plus 2.5 times the interquartile range) and were excluded from further analyses (Multimedia Appendix 1). On the basis of the reduced dataset, the estimated average compliance and retention increased slightly to 79.7% (95% CI 77.5-81.8) and 94% (95% CI 92.0-95.7), respectively.

The underlying true effects were heterogeneous, showing Q_104_=3398.31, *P<*.001, and Q_111_=666.94, *P<*.001, for compliance and retention, respectively. For compliance, the estimates of the between-study and between-group variance components were 50.9 (95% CI 22.4-89.4) and 33.3 (95% CI 19.7-58.2), respectively. Hence, a larger part of the total amount of heterogeneity in the underlying true outcomes was because of differences between studies (60%) as opposed to differences between groups (40%). The same pattern held for retention, with estimated between-study and between-group variance components of 0.015 (95% CI 0.006-0.028; 57% of total amount of heterogeneity) and 0.011 (95% CI 0.005-0.022; 43% of total amount of heterogeneity), respectively.

**Figure 3:**
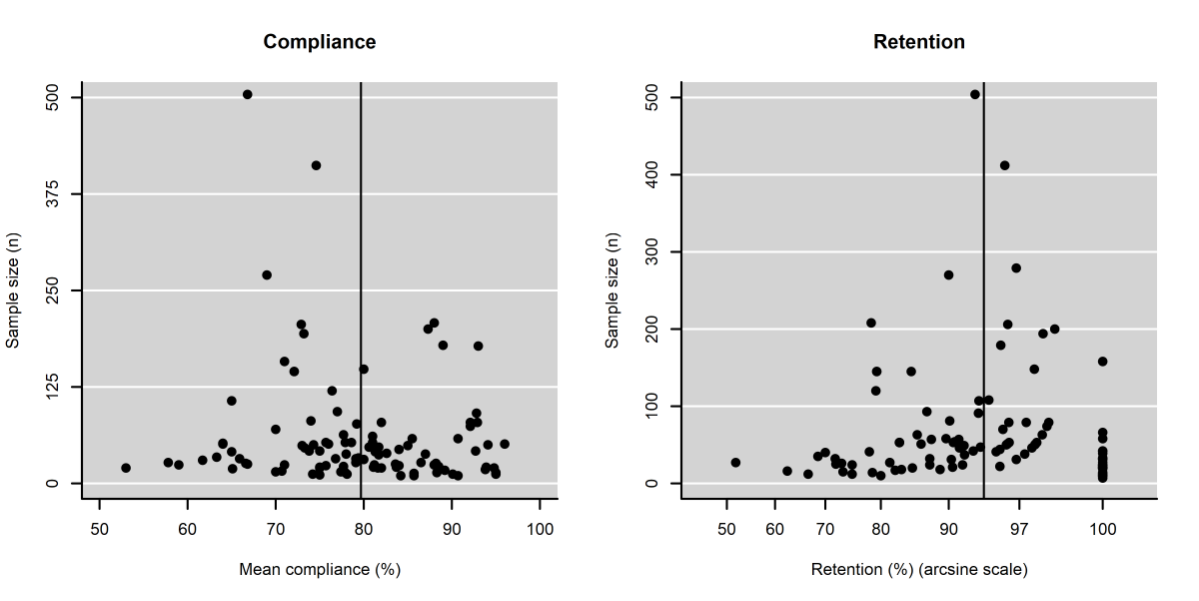
Funnel plots for compliance and retention.

Visual inspection of the funnel plots did not reveal any marked asymmetry (Figure 3). Moreover, the regression test for funnel plot asymmetry was not significant for either outcome (*P*=.24 and *P*=.84, respectively).

### Meta-Regression Analyses of the Sample Characteristics

The results of the meta-regression analyses of the sample characteristics are shown in Table 2. For some continuous predictors, the distribution of the predictor included some extremely large or low values. In such cases, we restricted the analysis to a range that excluded such extreme values. Scatterplots of the unrestricted and the restricted data (where applicable) are provided as part of Multimedia Appendix 1.

The analyses revealed significant relationships between some of the characteristics of the participants and the mean compliance and retention rates. Specifically, the proportion of women in ESM studies was found to be a significant predictor of both compliance (*P<*.001) and retention (*P*=.006), with estimated compliance and retention levels increasing by 18.1% and 11.9% points, respectively, when comparing a sample constituted exclusively of female participants with a sample composed exclusively of male participants. Second, the clinical status of the participants was also found to be a significant predictor of compliance and retention (*P<*.001). In particular, mean compliance and retention rates of samples of individuals without a psychiatric condition were estimated to be 10.8% and 9.5% points, respectively, higher when compared with samples of individuals with a psychotic disorder. Contrary to our expectations based on previous research, the mean age of the samples did not exhibit a significant relationship with compliance (*P*=.08) nor retention (*P*=.35).

**Table 2:** Results of the meta-regression analyses of the sample characteristics.

Sample characteristics			k	Estimate	SE	*P* value	95% CI	Q_M_ test (*df*)	R² (%)	
									Study	Group
**Compliance**										
	**Age**		98					—^a^	34	0
		Intercept		85.65	3.44		78.91-92.39			
		Beta		−0.18	0.1	.08	−0.38 to 0.02			
	**Gender (% female)**		99					—	0	44
		Intercept		68.41	2.51		63.49-73.33			
		Beta		0.18	0.04	<.001	0.11-0.25			
	**Clinical status**		105					41.48 (5)	0	54
		Intercept (HC^b^)		82.61	1.53		79.61-85.6			
		Beta (GP^c^)		−1.55	2.6	.55	−6.64 to 3.54			
		Beta (HR^d^)		−1.67	2.36	.48	−6.30 to 2.96			
		Beta (MDD^e^)		−0.77	1.8	.67	−4.31 to 2.76			
		Beta (BD^f^)		0.57	2.44	.82	−4.21 to 5.36			
		Beta (PD^g^)		−10.77	1.75	<.001	−14.2 to −7.34			
**Retention**										
	**Age**		102					—	0	42
		Intercept		1.382	0.067		1.250-1.514			
		Beta		−0.00	0.002	.35	−0.006-0.002			
	**Gender (% female)**		107					—	12	0
		Intercept		1.183	0.055		1.075-1.290			
		Beta		0.002	0.001	<.01	0.001-0.004			
	**Clinical status**		112					26.27 (5)	0	41
		Intercept (HC)		1.405	0.031	—	1.344-1.466			
		Beta (GP)		−0.081	0.047	.09	−0.173 to 0.011			
		Beta (HR)		−0.123	0.064	.06	−0.249 to 0.004			
		Beta (MDD)		−0.035	0.041	.39	−0.114 to 0.045			
		Beta (BD)		−0.098	0.064	.13	−0.224 to 0.028			
		Beta (PD)		−0.192	0.039	<.001	−0.268 to −0.116			

^a^Not applicable.

^b^HC: healthy control.

^c^GP: general population.

^d^HR: high risk for a severe mental disorder.

^e^MDD: major depressive disorder.

^f^BD: bipolar disorder.

^g^PD: psychotic disorder.

### Meta-Regression Analyses of the Design Characteristics

**Figure 4:**
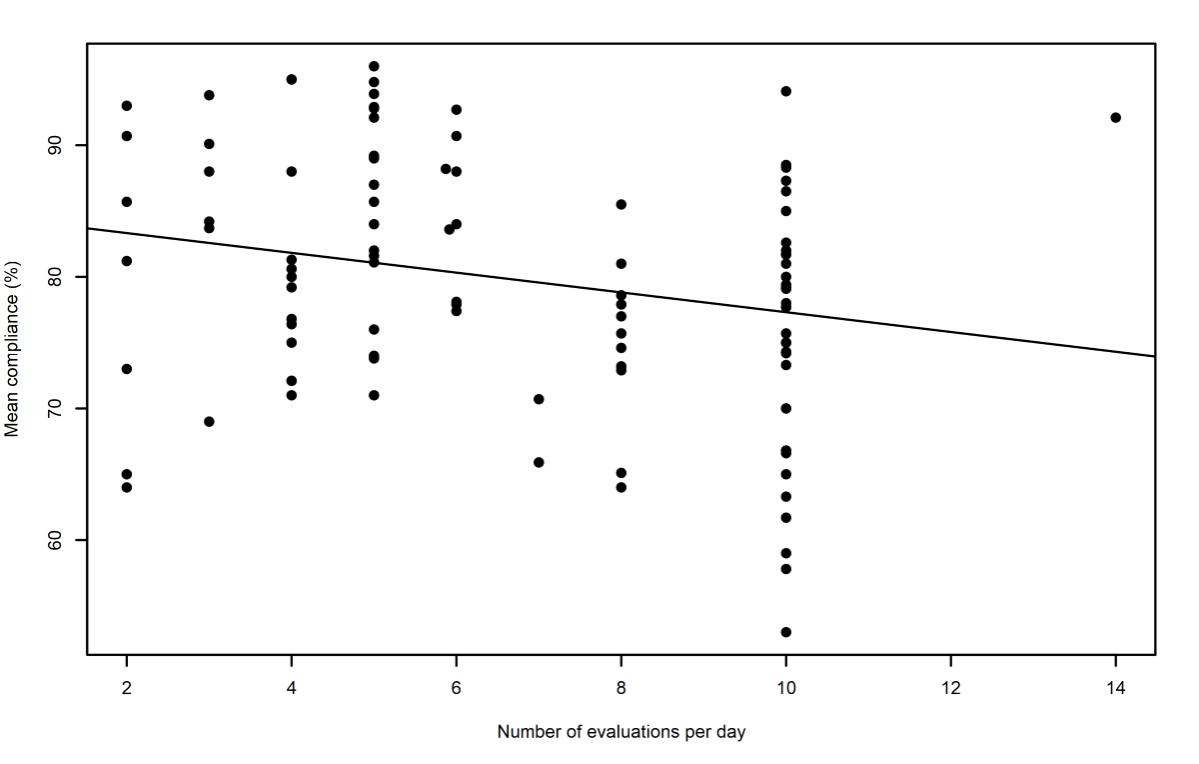
Graphical representation of the relationship between the compliance of experience sampling method studies and the frequency of daily self-evaluations.

The results of the meta-regression analyses of the design characteristics are shown in Table 3. The analyses revealed significant relationships between some of the design characteristics and compliance but not with retention. First, the number of evaluations per day was found to be a significant predictor of compliance (*P*=.008). To illustrate, mean compliance is estimated to fall by 8% points when comparing a follow-up involving 2 evaluations per day with a follow-up involving 10 evaluations per day (Figure 4).

Second, the duration of the time interval between successive evaluations within a day was also found to be a significant predictor of compliance (*P*=.02), with an estimated decrease in mean compliance by 10.8% points when comparing time intervals of 240 min with time intervals of 60 min. Third, relying on fixed sampling is predicted to yield a mean compliance that is 6.7% points higher (*P*=.02) compared with more conventional semirandom sampling (which did not differ from random sampling, *P*=.78). Fourth, the use of Web-based or mixed data collection method (ie, using different devices or platforms) was found to be a significant predictor of compliance (*P*=.03) compared with the use of PDAs, with an estimated decrease in mean compliance by 14% points and 16.5% points, respectively. Finally, the value of the incentives was found to significantly predict compliance (*P*=.02), with an estimated increase of 8.8% points in mean compliance when comparing the use of €20 incentives with the use of €200 incentives.

**Table 3:** Results of the meta-regression analyses of the design characteristics.

Design characteristics			k	Estimate	SE	*P* value	95% CI	Q_M_ test (*df*)	R² (%)	
									Study	Group
**Compliance**										
	**Evaluations**		104					—^a^	19	0
		Intercept		86.23	2.75		80.84-91.61			
		Beta		−0.99	0.38	<.01	−1.73 to −0.25			
	**Days**		103					—	1	0
		Intercept		78.69	1.86		75.04-82.34			
		Beta		0.14	0.18	.43	−0.21 to 0.49			
	**Hours/day**		76					—	36	0
		Intercept		74.8	10.41		54.39-95.21			
		Beta		0.28	0.76	.71	−1.21 to 1.78			
	**Duration between evaluations**		71					—	51	0
		Intercept		71.43	3.4		64.76-78.10			
		Beta		0.06	0.02	.02	0.01-0.11			
	**Items**		83					—	0	0
		Intercept		81.96	2.39		77.27-86.65			
		Beta		−0.15	0.1	.14	−0.34 to 0.05			
	**Sampling scheme**		103					6.78	22	0
		Intercept (semirandom)		78.5	1.64		75.27-81.72			
		Beta (random)		−0.63	2.29	.78	−5.13 to 3.86			
		Beta (fixed)		6.7	2.95	.02	0.90-12.50			
	**Data collection method**		105					14.98	27	0
		Intercept (PDA^b^)		81.14	1.38		78.45-83.84			
		Beta (paper-pencil)		−2.90	2.24	.20	−7.29 to 1.49			
		Beta (calls)		6.89	4.75	.15	−2.43 to 16.20			
		Beta (SMS)		−0.91	6.06	.88	−12.79 to 10.97			
		Beta (voicemail)		−12.64	8.19	.12	−28.69 to 3.41			
		Beta (Web-based)		−13.99	6.49	.03	−26.70 to −1.27			
		Beta (mixed)		−16.5	7.79	.03	−31.77 to −1.23			
	**Scale type**		102					0.28^c^	7	0
		Intercept (LS^d^)		79.03	1.45		76.19-81.87			
		Beta (VAS^e^)		−0.84	3.45	.81	−7.60 to 5.93			
		Beta (mixed)		0.98	2.48	.69	−3.87 to 5.83			
	**Incentives**		43					—	23	0
		Intercept		75.36	2.23		70.99-79.73			
		Beta		0.04	0.02	.02	0.01-0.09			
**Retention**										
	Evaluations		111						0	1
		Intercept		1.275	0.053		1.171-1.379			
		Beta		0.007	0.007	.34	−0.007 to 0.020			
	**Days**		109					—	0	0
		Intercept		1.329	0.036		1.259-1.399			
		Beta		−0.000	0.004	.96	0.007-0.007			
	**Hours/day**		87					—		
		Intercept		1.358	0.186		0.994-1.722			
		Beta		−0.001	0.014	.92	−0.028 to 0.025			
	**Duration between evaluations**		86					—	2	17
		Intercept		1.36	0.06		1.243-1.478			
		Beta		−0.000	0	.71	−0.001 to 0.001			
	**Items**		92					—	0	2
		Intercept		1.274	0.044		1.188-1.360			
		Beta		0.002	0.002	.35	−0.00 to 0.01			
	**Sampling scheme**		111					0.17^c^	0	0
		Intercept (semirandom)		1.322	0.031		1.263-1.382			
		Beta (random)		−0.007	0.045	.88	−0.095 to 0.082			
		Beta (fixed)		0.018	0.058	.76	−0.095 to 0.131			
	**Data collection method**		112					7.22^c^	7	0
		Intercept (PDA)		1.342	0.026		1.291-1.393			
		Beta (paper-pencil)		−0.039	0.043	.36	−0.124 to 0.046			
		Beta (calls)		−0.123	0.114	.28	−0.346 to 0.101			
		Beta (SMS)		0.082	0.121	.50	−0.155 to 0.318			
		Beta (Web-based)		−0.153	0.089	.09	−0.328 to 0.022			
		Beta (mixed)		0.229	0.164	.16	−0.093 to 0.550			
	**Scale type**		111					1.55^c^	0	0
		Intercept (LS)		1.3	0.026		1.248-1.352			
		Beta (VAS)		0.062	0.07	.37	−0.074 to 0.198			
		Beta (mixed)		0.047	0.045	.30	−0.042 to 0.135			
	**Incentives**		52					—	0	19
		Intercept		1.272	0.041		1.193-1.352			
		Beta		0	0	.62	−0.001 to 0.001			

^a^Data not applicable.

^b^PDA: personal digital assistant.

^c^Not significant.

^d^LS: Likert scale.

^e^VAS: visual analogue scale.

## Discussion

The aim of the present meta-analysis was to investigate compliance and retention rates in ESM studies including subjects across the spectrum of severe mental disorders and to examine how these outcomes are related to various person characteristics and design aspects. First, we found relatively high mean levels of compliance (ie, 78.7%) and retention (ie, 93.1%) across the included ESM studies. This is in line with previous findings in individuals with chronic pain [16] and substance users [33], supporting the feasibility and acceptability of ESM in mental health research. Second, we were also able to identify several sample and design characteristics that appear to be related to both the compliance and retention rate in ESM studies.

### Influence of the Sample Characteristics

Both the gender composition and the clinical status of the groups were found to predict the degree of compliance and retention in ESM studies. First, the proportion of male participants within a sample was negatively associated with compliance, supporting similar findings in adolescents [15] and adult samples [14,32,47]. Second, as reported previously in the literature [32,48], individuals with a psychotic disorder exhibited significantly lower compliance and retention rates compared with the other groups. In contrast, we did not find differences in the mean compliance and retention rates in samples at risk for a psychiatric condition and in individuals with mood disorders compared with healthy control or general population samples. This result is not in line with previous findings suggesting that greater negative affect in adolescents [15] and higher depressive symptoms in young adults [47] predicts lower compliance with ESM. The lower compliance in individuals with a psychotic disorder may be because of the inclusion of more severely ill people (eg, during acute phases of psychosis) or because of the presence of more severe cognitive impairments in individuals with a psychotic disorder compared with a major depressive [49] or bipolar disorder [50]. Finally, contrary to previous studies [16,32], we did not observe a significant association between the mean age of the samples and compliance. This could be because of a difference in the nature of the sample, with Morren et al review [16] focusing specifically on chronic pain patients, or to a difference in the nature of the study design, with Rintala et al [32] relying exclusively on paper-and-pencil assessment schemes. Thus, while younger samples were found to be less compliant when ESM assessments were conducted using a paper-and-pencil approach, the emergence of electronic devices in ESM research together with the current mobile phone use habits in young individuals [51] may have facilitated and increased the feasibility of ESM studies in younger samples.

In sum, ESM studies in individuals with a psychotic disorder or in samples with a higher proportion of male participants are at risk for lower compliance and retention rates. To increase compliance and retention, researchers could engage in procedures that aim to maintain the compliance of the participants as described in the review of Morren et al [16], such as sending reminders, providing a more extensive briefing, or contacting the participants regularly by phone to increase motivation. However, Jones et al [33] did not find any difference in compliance between studies mentioning a preliminary training of the participants for ESM and the ones not mentioning it. These methods may thus not be sufficient to improve compliance. Therefore, the potentially higher loss of data should also be taken into account in the sample size calculation preceding any ESM study investigating individuals with these characteristics.

### Influence of the Design Characteristics

We also found a number of design characteristics that were associated with the compliance and retention rates. First, the number of evaluations per day was associated with compliance levels in the ESM studies. On average, for each additional evaluation per day, mean compliance is predicted to fall by approximately 1% point. However, a lower compliance rate with a higher number of evaluations may still result in more data points. For example, according to our results, an ESM study involving 8 evaluations per day would result in an estimated mean of 6.18 completed evaluations/day, whereas a sampling frequency of 7 evaluations per day would result in only 5.48 evaluations/day. This result does not corroborate the findings of previous single studies investigating samples with different characteristics [17,33], which could be explained by the potential lack of statistical power inherent in single studies. In addition, the severity of the psychiatric disorders under study in the current meta-analysis compared with the aforementioned conditions might play a role in this discrepancy of results. For instance, individuals with severe mental disorders might be more reactive to the repetition of self-evaluations through the requirement of larger cognitive efforts to self-evaluate or the experience of a greater affective reactivity to the follow-up compared with individuals with milder conditions.

Second, the current meta-analysis found no significant association between the number of data collection days and the compliance and retention rates. This result corroborates the absence of an effect of study duration on compliance observed in substance users [33]. This finding is also in line with an ESM study in patients with schizophrenia [52], which reported that missing data were not associated with the number of assessment days in the study. These findings are particularly worth emphasizing when considering the current common practice in ESM research in severe mental disorders. Indeed, in the current review, most studies relied on relatively intensive (ie, median number of evaluations per day, 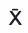
=7.5 evaluations) and short (ie, median duration of ESM studies, 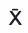
=7 days) assessment schemes. Given the current findings, together with the observation of a beneficial effect of longer intervals between successive evaluations on mean compliance, it may be worthwhile for researchers and practitioners to favor longer protocols with less intensive assessment frequencies to maximize compliance to ESM while collecting the same amount of data. Some statistical approaches (eg, time-lagged analyses or network analyses) [53] could, however, require a sufficient number of evaluations at the day level.

Third, our analyses revealed an association between the ESM sampling strategy and the compliance and retention rate, with fixed sampling schemes resulting on average in higher compliance and retention rates. Although this seems to favor fixed over random sampling schemes to improve the quantity of the data, the choice is not so simple. For instance, Husky et al [54] used a fixed sampling scheme and reported that participants were more likely to be alone over the duration of the ESM study, an observation that “may reflect the choice of participants to be in a quiet environment or to otherwise isolate themselves when completing electronic interviews.” In other words, a fixed sampling scheme allows participants to plan their daily tasks in accordance with the scheduled assessment times, which may increase compliance rates but potentially at the cost of lower ecological validity and increased bias. A random assessment scheme would avoid this problem, but, as argued by Piasecki et al [55], random time sampling may be perceived as more burdensome by study participants, thus potentially leading to lower compliance because of the respondents not knowing when the next assessment will occur. As such, if both sampling schemes present respective advantages, the current meta-analysis cannot clearly establish the optimal choice regarding this design characteristic. Therefore, this choice should be based on the requirements of the scientific question under study.

Fourth, we found a positive association between the value of the incentives and the compliance rates in ESM studies, similar to what was reported by Morren et al [16] in chronic pain patients. In contrast, Jones et al [33] did not find any effect of tying the amount of the incentives to the compliance rates (eg, providing an incentive per filled out report). However, it is worth noting that we did not consider the administration mode of the incentives, nor the value of the incentives per evaluation, but only the total value of the incentives provided to the participants at the end of the study.

Finally, no significant differences in compliance or retention rates were found between studies using a PDA compared with paper-and-pencil diaries. A similar result was recently reported in a meta-analysis of ESM studies in substance users [33]. In addition, the number of items within the ESM questionnaire was not significantly associated with compliance or retention, which contradicts previous research that found a lower number of items to be associated with higher compliance rates [16]. One reason for this discrepancy may be the lack of transparency about the actual number of items used in an ESM questionnaire. As argued by Morren et al [16], most studies only report the items that they have included in the analyses and hence may fail to report the actual number of items used in the entire questionnaire. This lack of transparency necessarily undermines the reliability of the analyses.

In fact, this point underscores a more general lack of clarity in the description of the methods used in ESM research, an issue previously underlined by Morren et al [16] and Jones et al [33]. In our sample, 73% of the studies reported both compliance and retention rates, which is definitely higher than the proportion observed in the review by Morren et al, where only 25% of the studies reported both these indexes [16]. However, it is necessary to point out that (1) this relatively high proportion of studies reporting compliance and retention rates in the current review is likely to be an overestimation as our inclusion criteria required at least 1 of these indexes to be reported and; (2) if mean compliance was reported in 82% of the studies, the corresponding variance was only reported in 50% of the studies. We, therefore, argue that ESM studies should clearly disclose all aspects of the protocol while systematically providing the standard statistical indexes (ie, mean and variance of the compliance rate and the retention rate) to allow an assessment of the quality of the data collection procedures.

### Recommendations

Overall, this systematic review and meta-analysis demonstrate that both the characteristics of the samples under study and the design of ESM studies may influence compliance and retention rates in ESM research. On the basis of these findings, we propose the following recommendations:

1. There is evidence that compliance and retention rates depend on the characteristics of the individuals under investigation. Samples of individuals with psychosis and a higher number of male participants appear to have a higher risk of lower compliance and retention. The potentially higher loss of data should be taken into account in the sample size calculation preceding any ESM study investigating individuals with these characteristics.
2. The evidence also suggests that the degree of compliance depends on various design choices in ESM studies.
  - A higher number of evaluations per day and smaller time intervals between successive evaluations are associated with lower compliance, whereas this is not the case for the number of days in an ESM study. Therefore, it may be worthwhile to decrease the number of evaluations while increasing the number of days, as such obtaining a similar number of data points while maximizing compliance.
  - The total amount of the incentive was associated with better compliance. Therefore, increasing the amount of the incentive may have a beneficial effect on the compliance of the participants with an ESM study.
3. The relative lack of transparency in reporting ESM protocols is likely to undermine the replicability of ESM studies and the assessment of their feasibility in severe mental disorders.
  - We recommend disclosing clearly all aspects of the procedures used in a given ESM study, regardless of their relevance for a given study, including but not limited to the actual number of ESM items participants answered, the amount of time between a signal and the answer of a participant that experimenters used to define compliance with a momentary evaluation, and any exclusion reasons, especially if experimenters exclude participants based on a predefined minimal mean compliance level.
  - We advise to report both the compliance mean level and the related SD, and the retention rate. When possible, this information should be provided at the group level.

### Limitations

This is the first review to systematically investigate predictors of compliance and retention rates in ESM research in severe mental disorders. However, despite its strengths, this review is not without limitations. First, the inconsistent report of essential information on the design of the ESM studies is likely to have introduced statistical errors in the estimation of the associations.

Second, compliance and retention rates are differently operationalized across studies in the literature. For compliance, evaluations are considered unanswered if the participants responded after 15 min following the trigger in some studies [11], whereas others used shorter time windows [56]. Concomitantly, subjects may only be retained for the analysis if they exceed a certain minimal compliance threshold [11], a threshold that also varies across studies. Thus, as the calculation of both these central indexes is not standardized in current practice in ESM research, the results might also reflect the heterogeneity of the experimenters’ methodological decisions.

Finally, it would have been of interest to examine to what degree potential participants are willing to participate in a study using ESM as a data collection method in the first place (and whether this is associated with certain participant or design characteristics). A brief search of the literature revealed considerable heterogeneity in reported *acceptance rates* across studies investigating clinical populations, varying from 38% in a group of patients with acute psychotic symptoms [57] to 96% in patients with schizophrenia [52], and from 67% to 97% in patient groups with an affective disorder [58,59]. Unfortunately, this type of information is not regularly reported in the literature and, if so, in even less standardized ways than compliance and retention rates. We were therefore unable to investigate this outcome in a systematic manner as part of this meta-analysis.

### Conclusions

This meta-analysis constitutes a first step toward the optimization of ESM research. Compliance and retention were associated with the gender and clinical status of the participants. Compliance, but not retention, was also associated with a number of design characteristics. In particular, compliance was lower with higher sampling frequencies but not with the duration of ESM studies, a finding that stands in contrast with current practices in ESM research. This review also demonstrates that ESM studies can be carried out in mental health research, but the quality of the data collection may depend upon a number of factors related to the design of the studies and samples under investigation that need to be considered when designing such protocols.

## Appendix

Multimedia Appendix 1 Supplementary material.

## Abbreviations

BD: bipolar disorder
EMA: ecological momentary assessment
ESM: experience sampling method
GP: general population
HC: healthy control
HR: high risk for a severe mental disorder
LS: Likert scale
MDD: major depressive disorder
PD: psychotic disorder
PDA: personal digital assistant
PRISMA-P: Preferred Reporting Items for Systematic Review and Meta-Analysis Protocols
VAS: visual analogue scale
