# Supplementary material for: Compliance and Retention With the Experience Sampling Method Over the Continuum of Severe Mental Disorders: Meta-Analysis and Recommendations
Source: J Med Internet Res. 2019 Dec 6;21(12):e14475. doi: 10.2196/14475 (PMC6925392; doi:10.2196/14475)
Supplement: Multimedia Appendix 1 [file jmir_v21i12e14475_app1.docx]

*Supplementary materials for:*

Compliance and retention with the Experience Sampling Method over the continuum of severe mental disorders: Meta-analysis and recommendations

*Concept plan of the search strategy*

| N0 | Query |
| --- | --- |
| 1 | (Major depressive disorder) AND (((((Momentary assessment) OR Experience sampling method) OR Ecological momentary assessment) OR Ambulatory study) OR Diary study) |
| 2 | (Depression) AND (((((Momentary assessment) OR Experience sampling method) OR Ecological momentary assessment) OR Ambulatory study) OR Diary study) |
| 3 | (Bipolar disorder) AND (((((Momentary assessment) OR Experience sampling method) OR Ecological momentary assessment) OR Ambulatory study) OR Diary study) |
| 4 | (Psychotic disorder) AND (((((Momentary assessment) OR Experience sampling method) OR Ecological momentary assessment) OR Ambulatory study) OR Diary study) |
| 5 | (Psychosis) AND (((((Momentary assessment) OR Experience sampling method) OR Ecological momentary assessment) OR Ambulatory study) OR Diary study) |
| 6 | (Schizophrenia) AND (((((Momentary assessment) OR Experience sampling method) OR Ecological momentary assessment) OR Ambulatory study) OR Diary study) |

*Overlapping samples*

Studies that fulfilled the inclusion criteria were examined for overlapping samples. Authors of studies performed at the same departments or catchment areas were asked for information on sample overlap. In case a paper reexamined multiple samples based on prior studies, these papers were excluded (i.e., only the original studies were included). This was the case for 5 papers [1–5]. The search also yielded a substantial number of papers that reported various findings based on the same sample of subjects, sometimes with slightly varying sample sizes. In this case, the report with the larger sample size was included. 57 studies were excluded for this reason [6–61]. If the sample size was identical for multiple papers reporting on the same sample, then the paper providing more information regarding the main outcomes was included (e.g., compliance at the group level versus compliance at the study level). 10 studies were excluded for this reason [62–71]. Finally, 79 studies fulfilled all inclusion criteria [72–149].

*Imputation of Missing Sampling Variances for Mean Compliance*

Let $\bar{p}_{i}$ denote the mean compliance in the $i$th group. The sampling variance of $\bar{p}_{i}$ was computed with $Var\left[ \bar{p}_{i} \right]=SD_{i}^{2}/n_{i}$, where $SD_{i}$ is the standard deviation of the compliance rates of the $n_{i}$ subjects in the *i*th group. Since $SD_{i}$ was not available for approximately half of the groups, we imputed missing $SD_{i}$ values based on the expected quadratic relationship between $\bar{p}_{i}$ and $SD_{i}$ (i.e., $SD_{i}$ must be 0 for $\bar{p}_{i}$ equal to 0 and 1 and will peak around $\bar{p}_{i}=0.5$). In particular, let $y_{i}=\ln\left[ SD_{i} \right]+1/(2(n_{i}-1))$ denote the log-transformed and bias-corrected $SD_{i}$ value for a particular group for which $SD_{i}$ is available (150). The sampling variance of $y_{i}$ is approximately $Var\left[ y_{i} \right]=1/(2(n_{i}-1))$. We then fitted a mixed-effects meta-regression to the $y_{i}$ values, with $\bar{p}_{i}$ and $\bar{p}_{i}^{2}$ as predictors. The results of this model are given below.

Mixed-Effects Model (k = 68; tau^2 estimator: REML)

tau^2 (estimated amount of residual heterogeneity): 0.0543 (SE = 0.0122)

tau (square root of estimated tau^2 value): 0.2330

I^2 (residual heterogeneity / unaccounted variability): 85.41%

H^2 (unaccounted variability / sampling variability): 6.85

R^2 (amount of heterogeneity accounted for): 68.42%

Test for Residual Heterogeneity:

QE(df = 65) = 316.9202, p-val < .0001

Test of Moderators (coefficients 2:3):

QM(df = 2) = 110.6069, p-val < .0001

Model Results:

estimate se zval pval ci.lb ci.ub

intrcpt 2.5738 1.0378 2.4801 0.0131 0.5398 4.6079 *

cmean 0.0378 0.0286 1.3219 0.1862 -0.0183 0.0939

I(cmean^2) -0.0005 0.0002 -2.4207 0.0155 -0.0009 -0.0001 *

---

Signif. codes: 0 ‘***’ 0.001 ‘**’ 0.01 ‘*’ 0.05 ‘.’ 0.1 ‘ ’ 1

As expected, the quadratic model was highly significant ($Q_{M}\left( df=2 \right)=110.61, p<.0001$) and highly predictive ($R^{2}=68.4\%$). Based on this model, we then imputed $y_{i}$ values for groups for which $y_{i}$ was unknown, but $\bar{p}_{i}$ was known. The figure below shows the observed $y_{i}$ values (⚫), the regression ‘line’ (i.e., curve) of the mixed-effects model above (with pointwise 95% CI bounds for the predicted value), and the imputed $y_{i}$ values (⚪). Missing $SD_{i}$ values were then computed with $\hat{SD}_{i}=exp[\hat{y}_{i}]$.


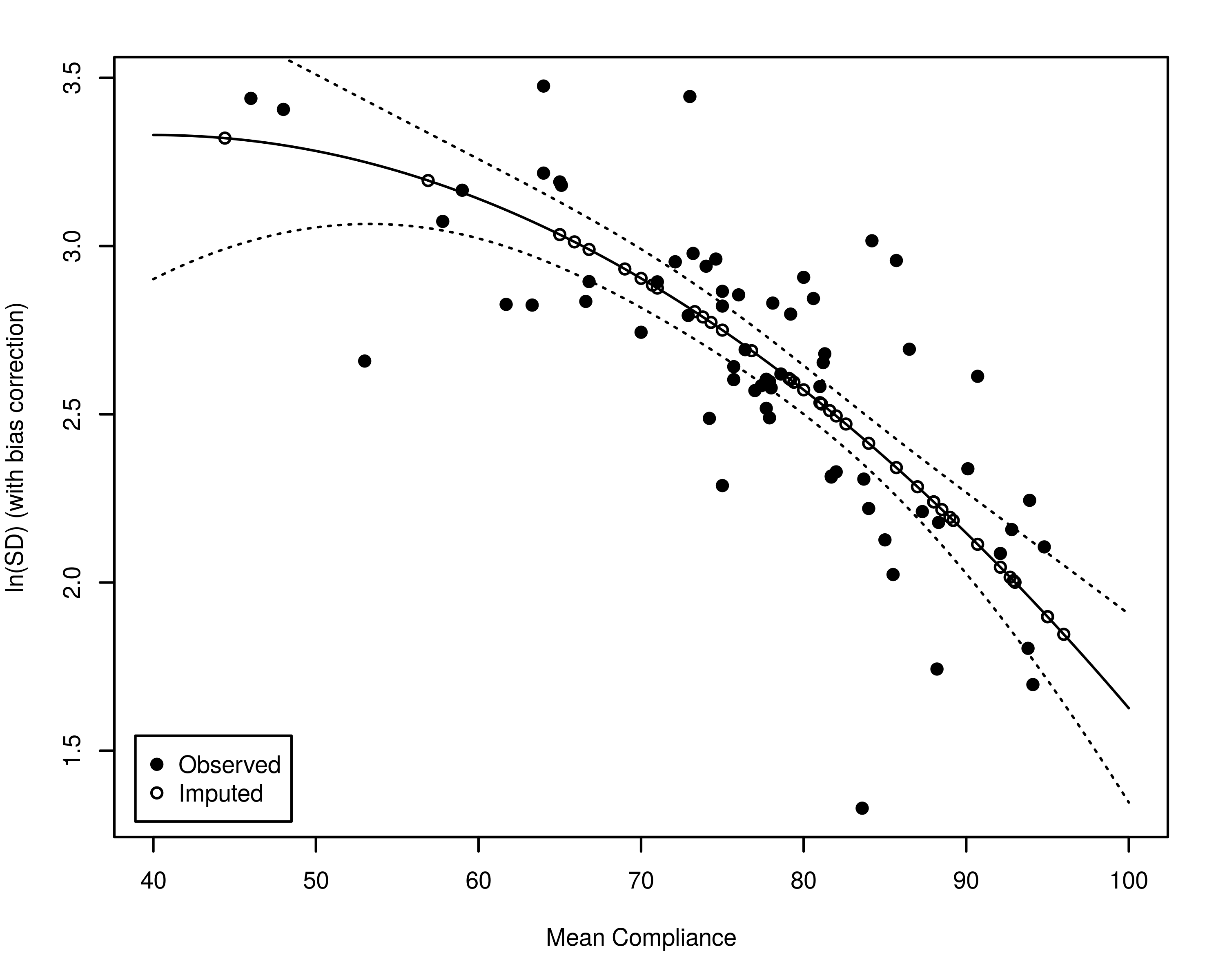


*Forest plots*


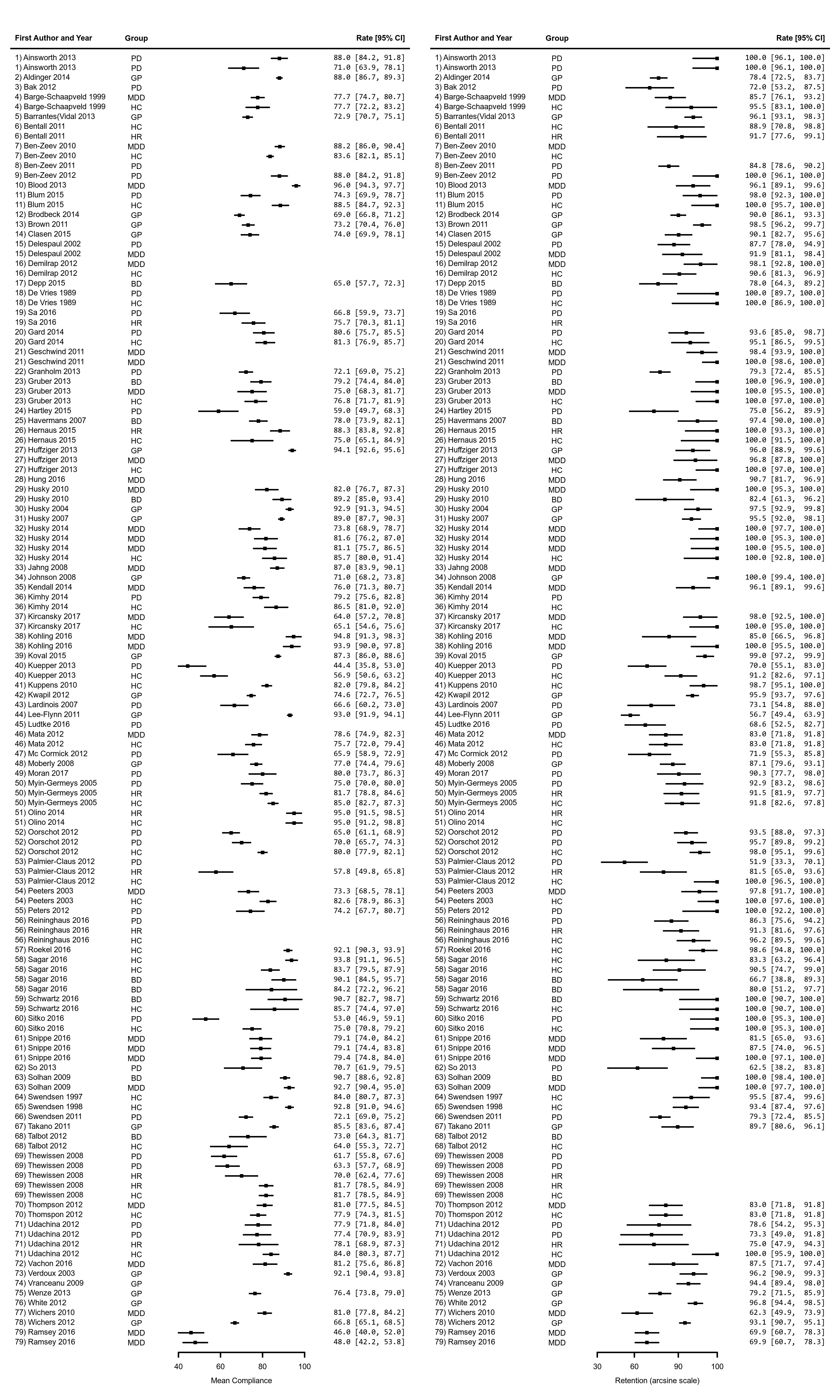


*Influential Studies*

For mean compliance, the plot of the Cook’s distances at the study level is shown below.


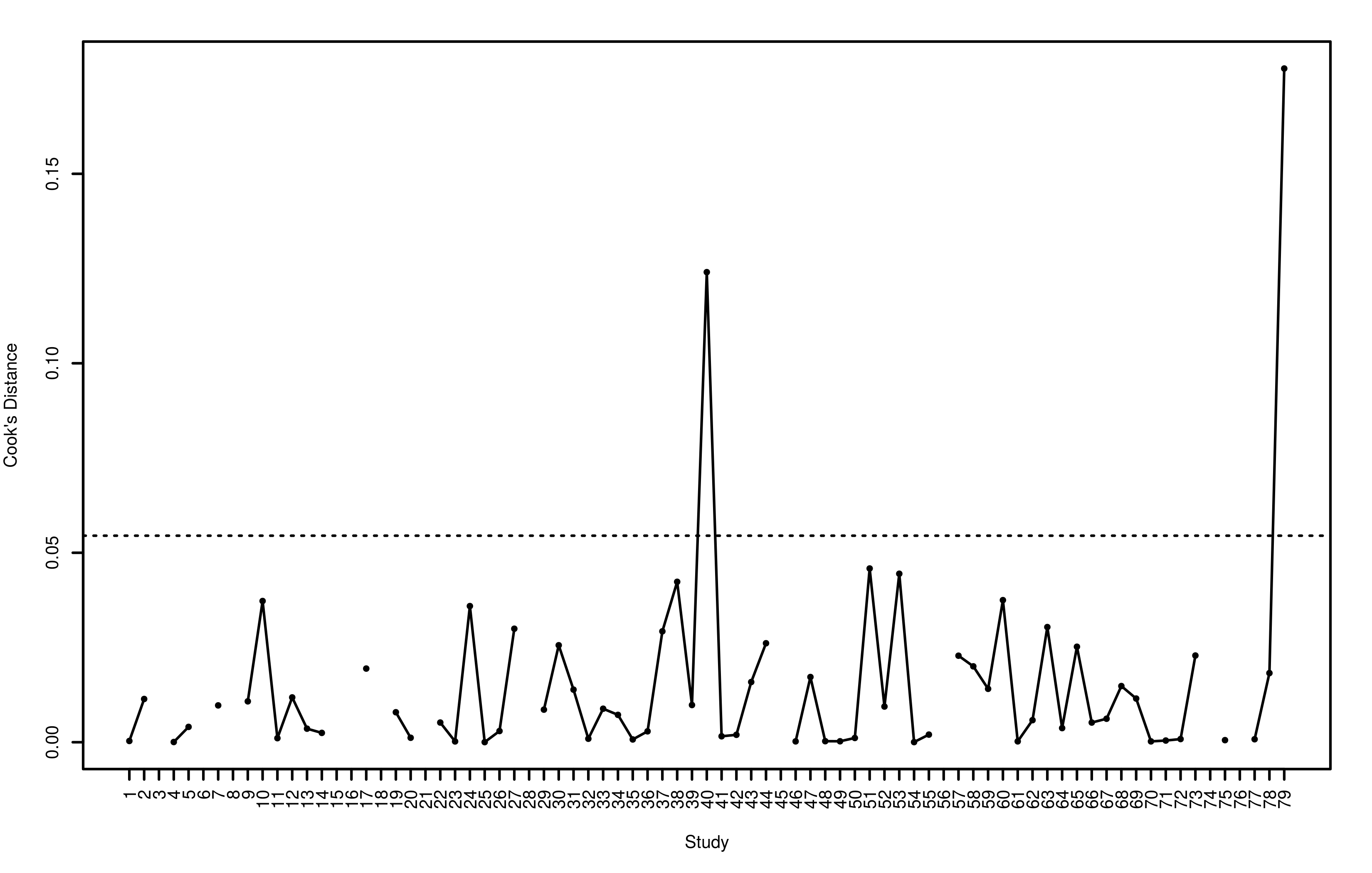


After removal of the influential studies (40 and 79), a plot of the Cook’s distances no longer showed the presence of overly influential studies.


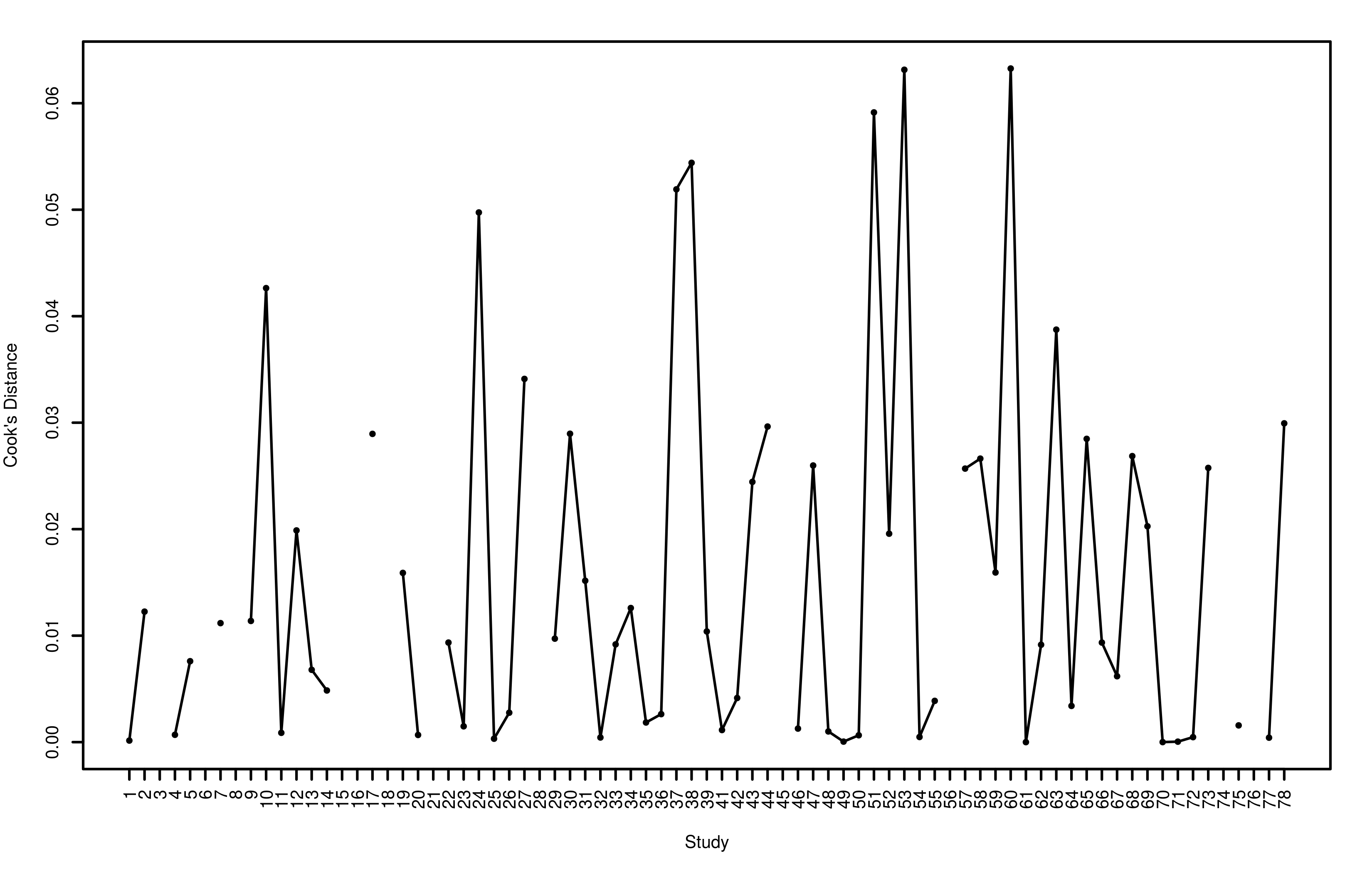


For retention, three studies (44, 77, 79) were found to be overly influential.


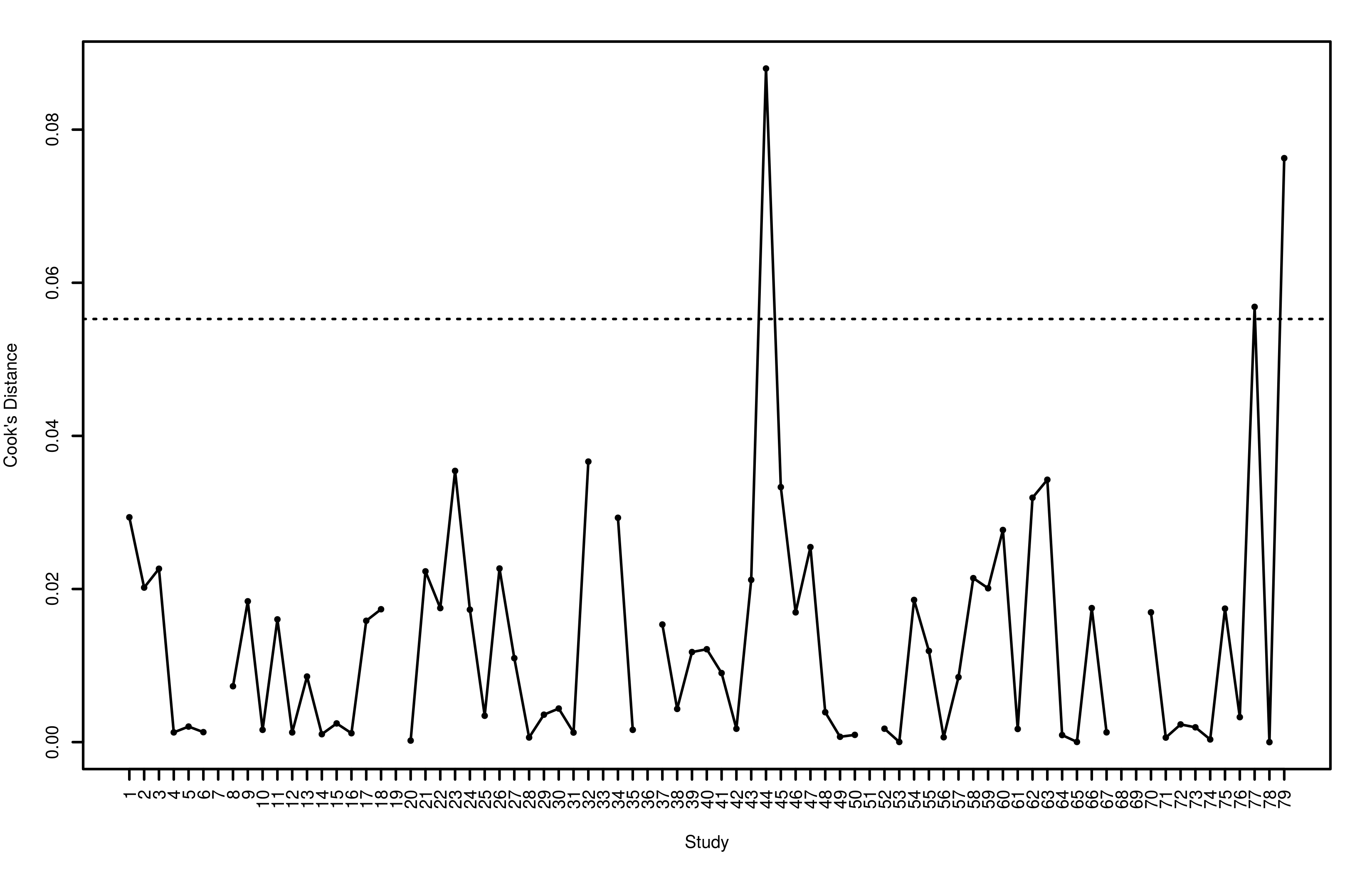


After their removal, a plot of the Cook’s distances no longer showed the presence of overly influential studies.


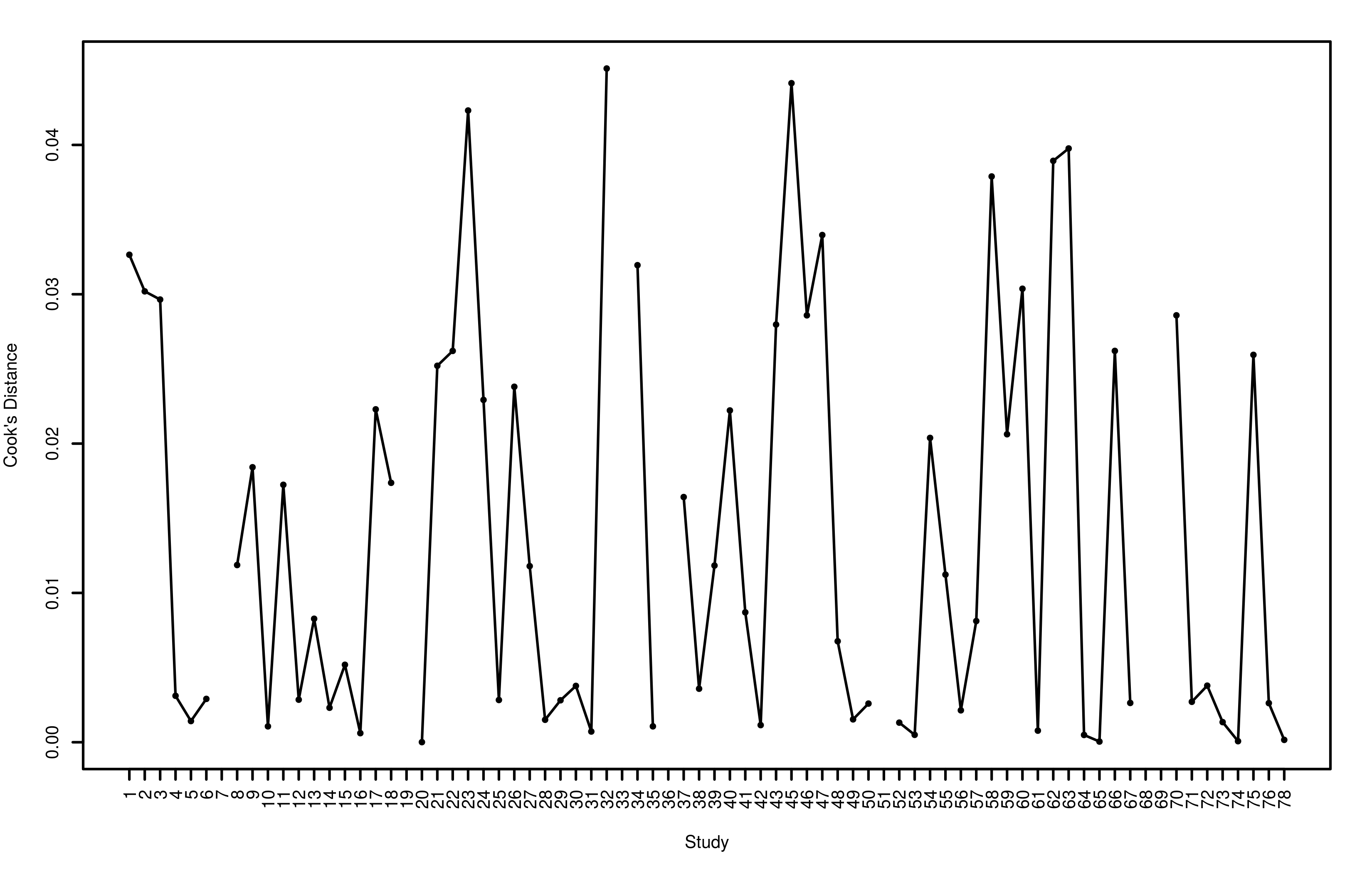


*Scatterplots of Moderators for Mean Compliance*

The figure below shows scatterplots of all quantitative moderators against mean compliance. When two plots are shown in the same row, then the data in the plot shown on the right is a subset of the full dataset, restricted to remove the influence of extreme values. These subsets were used in the analyses. The fitted lines from the meta-regression models are also shown.


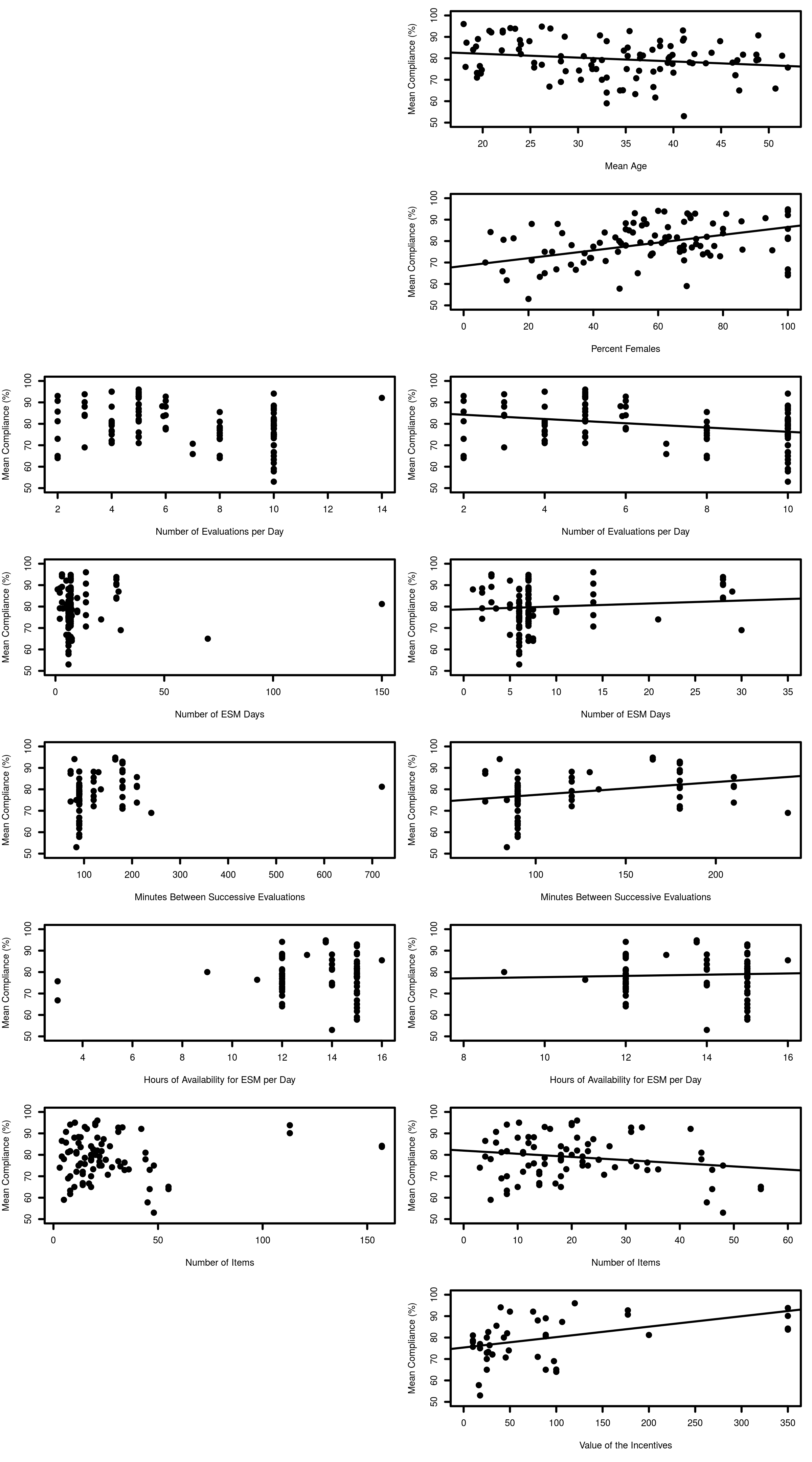


*Scatterplots of Moderators for Retention*

The figure below shows scatterplots of all quantitative moderators against retention (arcsine transformed). When two plots are shown in the same row, then the data in the plot shown on the right is a subset of the full dataset, restricted to remove the influence of extreme values. These subsets were used in the analyses. The fitted lines from the meta-regression models are also shown.


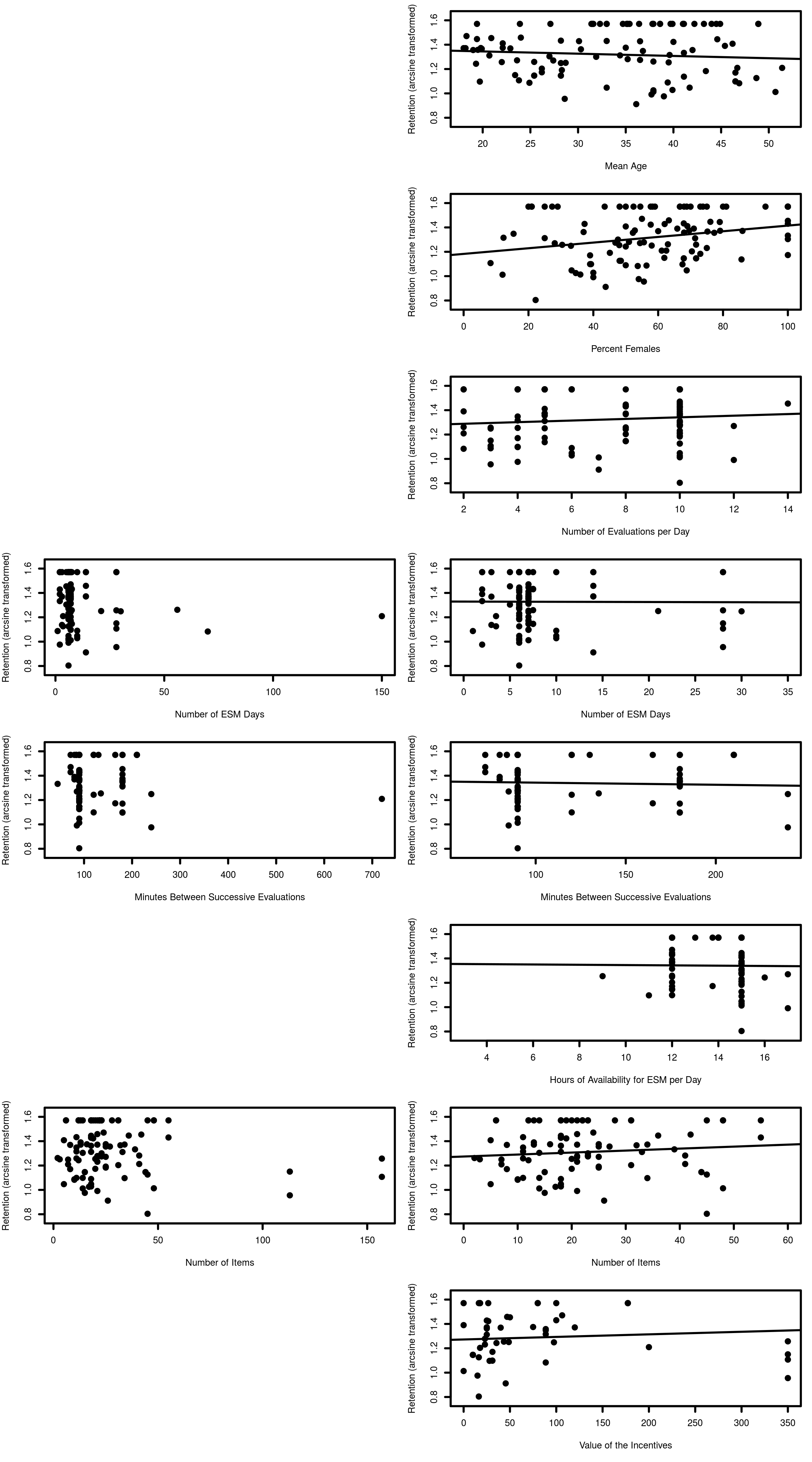


*Correlation between predictor variables*

For readers interested in the degree of correlation between the predictor variables examined in the meta-analysis, we provide a corresponding correlation matrix below. The values shown are pairwise correlations based on the available data for each pair of variables. For the categorical predictors, we dummy coded one or two of the relevant levels.


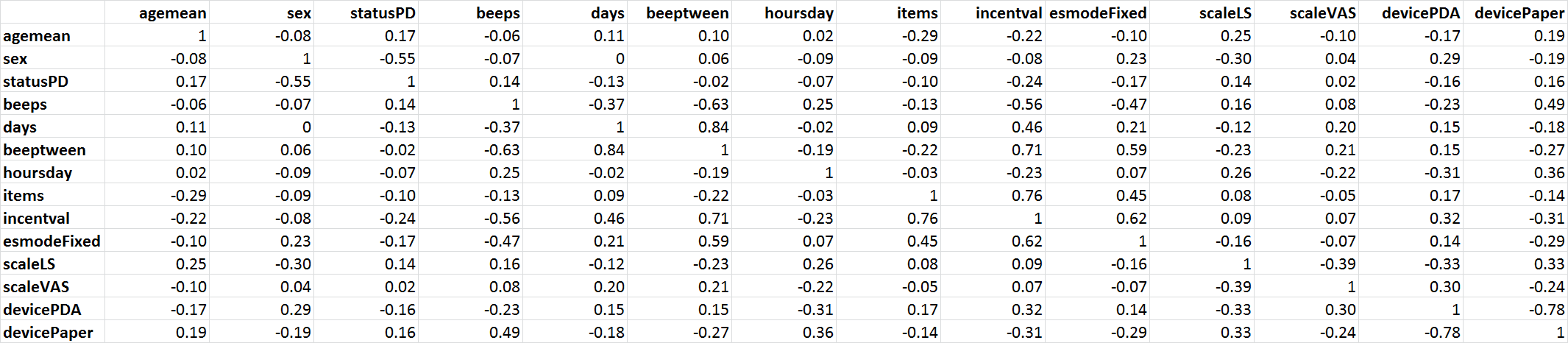


agemean = mean age of the sample

sex = percent females in the sample

statusPD = dummy for clinical status (1 = psychotic disorder, 0 = other)

beeps = evaluations per day

days = study days

beeptween = duration between evaluations

hoursday = hours/day

items = number of items

inventval = value of the incentive

esmodeFixed = dummy for sampling scheme (1 = Fixed, 0 = other)

scaleLS = dummy for scale type (1 = Likert scale, 0 = other)

scaleVAS = dummy for scale type (1 = visual analogue scale, 0 = other)

devicePDA = dummy for data collection method (1 = personal digital assistant, 0 = other)

devicePaper = dummy for data collection method (1 = paper-and-pencil, 0 = other)

*References (supplement)*

1. Lataster J, Thewissen V, Bak M, Lataster T, Lardinois M, Delespaul P, et al. Emotional experience and estimates of D2 receptor occupancy in psychotic patients treated with haloperidol, risperidone, or olanzapine: an experience sampling study. J Clin Psychiatry. 2011;72(10):1397–404.

2. Myin-Germeys I, Peeters F, Havermans R, Nicolson NA, DeVries MW, Delespaul P, et al. Emotional reactivity to daily life stress in psychosis and affective disorder: an experience sampling study. Acta Psychiatr Scand. 2003;107(2):124–131.

3. Oorschot M, Lataster T, Thewissen V, Bentall R, Delespaul P, Myin-Germeys I. Temporal dynamics of visual and auditory hallucinations in psychosis. Schizophr Res. 2012 Sep;140(1–3):77–82.

4. Oorschot M, Lataster T, Thewissen V, Lardinois M, Wichers M, van Os J, et al. Emotional experience in negative symptoms of schizophrenia—no evidence for a generalized hedonic deficit. Schizophr Bull. 2011;39(1):217–25.

5. Swendsen JD, Compagnone P. The expression of cognitive vulnerabilities for depression in daily life: a French-American study. Eur Psychiatry. 2000;15:22–28.

6. Collip D, Habets P, Marcelis M, Gronenschild E, Lataster T, Lardinois M, et al. Hippocampal volume as marker of daily life stress sensitivity in psychosis. Psychol Med. 2013;43(7):1377–87.

7. Collip D, Nicolson NA, Lardinois M, Lataster T, van Os J, Myin-Germeys I. Daily cortisol, stress reactivity and psychotic experiences in individuals at above average genetic risk for psychosis. Psychol Med. 2011 Nov;41(11):2305–15.

8. Collip D, Oorschot M, Thewissen V, Van Os J, Bentall R, Myin-Germeys I. Social world interactions: how company connects to paranoia. Psychol Med. 2011;41(5):911–21.

9. Collip D, Wigman JTW, van Os J, Oorschot M, Jacobs N, Derom C, et al. Positive emotions from social company in women with persisting subclinical psychosis: lessons from daily life. Acta Psychiatr Scand. 2014 Mar;129(3):202–10.

10. Collip D, van Winkel R, Peerbooms O, Lataster T, Thewissen V, Lardinois M, et al. COMT Val158Met–stress interaction in psychosis: role of background psychosis risk. CNS Neurosci Ther. 2011;17(6):612–9.

11. Frissen A, Lieverse R, Drukker M, Delespaul P, Lataster T, Myin-Germeys I, et al. Evidence that childhood urban environment is associated with blunted stress reactivity across groups of patients with psychosis, relatives of patients and controls. Soc Psychiatry Psychiatr Epidemiol. 2014;49(10):1579–87.

12. Geschwind N, Peeters F, Jacobs N, Delespaul P, Derom C, Thiery E, et al. Meeting risk with resilience: high daily life reward experience preserves mental health. Acta Psychiatr Scand. 2010;122(2):129–38.

13. Granholm E, Loh C, Swendsen J. Feasibility and Validity of Computerized Ecological Momentary Assessment in Schizophrenia. Schizophr Bull. 2007 May 4;34(3):507–14.

14. Hartmann JA, Wichers M, Menne-Lothmann C, Kramer I, Viechtbauer W, Peeters F, et al. Experience Sampling-Based Personalized Feedback and Positive Affect: A Randomized Controlled Trial in Depressed Patients. Franken IHA, editor. PLOS ONE. 2015 Jun 2;10(6):e0128095.

15. Havermans R, Nicolson NA, Berkhof J, deVries MW. Patterns of salivary cortisol secretion and responses to daily events in patients with remitted bipolar disorder. Psychoneuroendocrinology. 2011 Feb;36(2):258–65.

16. Henquet C, Rosa A, Delespaul P, Papiol S, Faňanás L, Van Os J, et al. COMT Val158Met moderation of cannabis‐induced psychosis: a momentary assessment study of “switching on”hallucinations in the flow of daily life. Acta Psychiatr Scand. 2009;119(2):156–60.

17. Henquet C, van Os J, Kuepper R, Delespaul P, Smits M, Campo J a., et al. Psychosis reactivity to cannabis use in daily life: an experience sampling study. Br J Psychiatry. 2010 Jun 1;196(6):447–53.

18. Jacobs N, Myin-Germeys I, Derom C, Delespaul P, Van Os J, Nicolson N. A momentary assessment study of the relationship between affective and adrenocortical stress responses in daily life. Biol Psychol. 2007;74(1):60–6.

19. Janssens M, Lataster T, Simons CJP, Oorschot M, Lardinois M, van Os J, et al. Emotion recognition in psychosis: No evidence for an association with real world social functioning. Schizophr Res. 2012 Dec 1;142(1):116–21.

20. Kimhy D, Delespaul P, Ahn H, Cai S, Shikhman M, Lieberman JA, et al. Concurrent measurement of “real-world” stress and arousal in individuals with psychosis: assessing the feasibility and validity of a novel methodology. Schizophr Bull. 2009;36(6):1131–9.

21. Kimhy D, Delespaul P, Corcoran C, Ahn H, Yale S, Malaspina D. Computerized experience sampling method (ESMc): assessing feasibility and validity among individuals with schizophrenia. J Psychiatr Res. 2006;40(3):221–30.

22. Kimhy D, Vakhrusheva J, Liu Y, Wang Y. Use of mobile assessment technologies in inpatient psychiatric settings. Asian J Psychiatry. 2014 Aug;10:90–5.

23. Kircanski K, Thompson RJ, Sorenson JE, Sherdell L, Gotlib IH. Rumination and worry in daily life: Examining the naturalistic validity of theoretical constructs. Clin Psychol Sci. 2015;3(6):926–39.

24. Kramer I, Simons C, Myin-Germeys I, Jacobs N, Derom C, Thiery E, et al. Evidence that genes for depression impact on the pathway from trauma to psychotic-like symptoms by occasioning emotional dysregulation. Psychol Med. 2012;42(2):283–94.

25. Kramer I, Simons CJ, Wigman JT, Collip D, Jacobs N, Derom C, et al. Time-lagged moment-to-moment interplay between negative affect and paranoia: new insights in the affective pathway to psychosis. Schizophr Bull. 2013;40(2):278–86.

26. Kwapil TR, Barrantes-Vidal N, Armistead MS, Hope GA, Brown LH, Silvia PJ, et al. The expression of bipolar spectrum psychopathology in daily life. J Affect Disord. 2011;130(1–2):166–70.

27. Lataster J, Myin-Germeys I, Wichers M, Delespaul PA, van Os J, Bak M. Psychotic exacerbation and emotional dampening in the daily life of patients with schizophrenia switched to aripiprazole therapy: a collection of standardized case reports. Ther Adv Psychopharmacol. 2011;1(5):145–151.

28. Lataster T, Valmaggia L, Lardinois M, van Os J, Myin-Germeys I. Increased stress reactivity: a mechanism specifically associated with the positive symptoms of psychotic disorder. Psychol Med. 2013 Jul;43(07):1389–400.

29. Marcelis M, Myin-Germeys I, Suckling J, Woodruff P, Hofman P, Bullmore E, et al. Cerebral tissue alterations and daily life stress experience in psychosis. Acta Psychiatr Scand. 2003;107(1):54–59.

30. Menne-Lothmann C, Jacobs N, Derom C, Thiery E, van Os J, Wichers M. Genetic and environmental causes of individual differences in daily life positive affect and reward experience and its overlap with stress-sensitivity. Behav Genet. 2012;42(5):778–86.

31. Morrens M, Krabbendam L, Bak M, Delespaul P, Mengelers R, Sabbe B, et al. The relationship between cognitive dysfunction and stress sensitivity in schizophrenia: A replication study. Soc Psychiatry Psychiatr Epidemiol. 2007 Apr;42(4):284–7.

32. Myin-Germeys I, Krabbendam L, Delespaul PAEG, Van Os J. Do life events have their effect on psychosis by influencing the emotional reactivity to daily life stress? Psychol Med. 2003 Feb;33(2):327–33.

33. Myin-Germeys I, Delespaul PA, deVries MW. Schizophrenia patients are more emotionally active than is assumed based on their behavior. Schizophr Bull. 2000;26(4):847.

34. Myin-Germeys I, Krabbendam L, Delespaul P, Van Os J. Sex differences in emotional reactivity to daily life stress in psychosis. J Clin Psychiatry. 2004;

35. Myin-Germeys I, Krabbendam L, Jolles J, Delespaul PA, van Os J. Are cognitive impairments associated with sensitivity to stress in schizophrenia? An experience sampling study. Am J Psychiatry. 2002;159(3):443–9.

36. Myin-Germeys I, Marcelis M, Krabbendam L, Delespaul P, van Os J. Subtle Fluctuations in Psychotic Phenomena as Functional States of Abnormal Dopamine Reactivity in Individuals at Risk. Biol Psychiatry. 2005 Jul;58(2):105–10.

37. Myin-Germeys I, Krabbendam L, Delespaul P, Van Os J. Can cognitive deficits explain differential sensitivity to life events in psychosis? Soc Psychiatry Psychiatr Epidemiol. 2003;38(5):262–8.

38. Myin-Germeys I, Nicolson NA, Delespaul PA. The context of delusional experiences in the daily life of patients with schizophrenia. Psychol Med. 2001;31(03):489–498.

39. Myin-Germeys I, Van Os J, Schwartz J, Stone AA, Delespaul P. Emotional reactivity to daily life stress in psychosis. Arch Gen Psychiatry. 2001;58:1137–44.

40. Oorschot M, Lataster T, Thewissen V, Wichers M, Myin-Germeys I. Mobile Assessment in Schizophrenia: A Data-Driven Momentary Approach. Schizophr Bull. 2012 May 1;38(3):405–13.

41. Peerbooms O, Rutten BPF, Collip D, Lardinois M, Lataster T, Thewissen V, et al. Evidence that interactive effects of COMT and MTHFR moderate psychotic response to environmental stress: COMT × MTHFR and stress sensitivity in psychosis. Acta Psychiatr Scand. 2012 Mar;125(3):247–56.

42. Sanchez AH, Lavaysse LM, Starr JN, Gard DE. Daily life evidence of environment-incongruent emotion in schizophrenia. Psychiatry Res. 2014 Dec;220(1–2):89–95.

43. Simons CJP, Hartmann JA, Kramer I, Menne-Lothmann C, Höhn P, van Bemmel AL, et al. Effects of momentary self-monitoring on empowerment in a randomized controlled trial in patients with depression. Eur Psychiatry. 2015 Nov;30(8):900–6.

44. Simons CJP, Wichers M, Derom C, Thiery E, Myin-Germeys I, Krabbendam L, et al. Subtle gene-environment interactions driving paranoia in daily life. Genes Brain Behav. 2009 Feb;8(1):5–12.

45. Takano K, Sakamoto S, Tanno Y. Ruminative self-focus in daily life: Associations with daily activities and depressive symptoms. Emotion. 2013;13(4):657–67.

46. Udachina A, Thewissen V, Myin-Germeys I, Fitzpatrick S, OʼKane A, Bentall RP. Understanding the Relationships Between Self-Esteem, Experiential Avoidance, and Paranoia: Structural Equation Modelling and Experience Sampling Studies. J Nerv Ment Dis. 2009 Sep;197(9):661–8.

47. Udachina A, Varese F, Myin-Germeys I, Bentall RP. The role of experiential avoidance in paranoid delusions: An experience sampling study. Br J Clin Psychol. 2014 Nov;53(4):422–32.

48. van Os J, Lataster T, Delespaul P, Wichers M, Myin-Germeys I. Evidence That a Psychopathology Interactome Has Diagnostic Value, Predicting Clinical Needs: An Experience Sampling Study. Jiménez-Murcia S, editor. PLoS ONE. 2014 Jan 23;9(1):e86652.

49. van Winkel M, Nicolson NA, Wichers M, Viechtbauer W, Myin-Germeys I, Peeters F. Daily life stress reactivity in remitted versus non-remitted depressed individuals. Eur Psychiatry. 2015 Jun;30(4):441–7.

50. van Winkel M, Peeters F, van Winkel R, Kenis G, Collip D, Geschwind N, et al. Impact of variation in the BDNF gene on social stress sensitivity and the buffering impact of positive emotions: Replication and extension of a gene–environment interaction. Eur Neuropsychopharmacol. 2014 Jun;24(6):930–8.

51. Walsh MA, Brown LH, Barrantes-Vidal N, Kwapil TR. The expression of affective temperaments in daily life. J Affect Disord. 2013 Feb;145(2):179–86.

52. Walsh MA, Royal A, Brown LH, Barrantes-Vidal N, Kwapil TR. Looking for bipolar spectrum psychopathology: identification and expression in daily life. Compr Psychiatry. 2012 Jul;53(5):409–21.

53. Wenze SJ, Gunthert KC, Forand NR. Influence of dysphoria on positive and negative cognitive reactivity to daily mood fluctuations. Behav Res Ther. 2007 May;45(5):915–27.

54. Wichers M, Aguilera M, Kenis G, Krabbendam L, Myin-Germeys I, Jacobs N, et al. The catechol-O-methyl transferase Val158Met polymorphism and experience of reward in the flow of daily life. Neuropsychopharmacology. 2008;33(13):3030–3036.

55. Wichers MC, Barge-Schaapveld D, Nicolson NA, Peeters F, De Vries M, Mengelers R, et al. Reduced stress-sensitivity or increased reward experience: the psychological mechanism of response to antidepressant medication. Neuropsychopharmacology. 2009;34(4):923–931.

56. Wichers M, Kasanova Z, Bakker J, Thiery E, Derom C, Jacobs N, et al. From Affective Experience to Motivated Action: Tracking Reward-Seeking and Punishment-Avoidant Behaviour in Real-Life. Dichter GS, editor. PLOS ONE. 2015 Jun 18;10(6):e0129722.

57. Wichers M, Lothmann C, Simons CJP, Nicolson NA, Peeters F. The dynamic interplay between negative and positive emotions in daily life predicts response to treatment in depression: A momentary assessment study: Emotional dynamics and future treatment response. Br J Clin Psychol. 2012 Jun;51(2):206–22.

58. Wichers M, Myin-Germeys I, Jacobs N, Peeters F, Kenis G, Derom C, et al. Genetic risk of depression and stress-induced negative affect in daily life. Br J Psychiatry. 2007;191(3):218–223.

59. Wichers M, Kenis G, Jacobs N, Myin-Germeys I, Schruers K, Mengelers R, et al. The psychology of psychiatric genetics: Evidence that positive emotions in females moderate genetic sensitivity to social stress associated with the BDNF Val^66^Met polymorphism. J Abnorm Psychol. 2008;117(3):699–704.

60. Wichers MC, Myin‐Germeys I, Jacobs N, Peeters F, Kenis G, Derom C, et al. Evidence that moment‐to‐moment variation in positive emotions buffer genetic risk for depression: a momentary assessment twin study. Acta Psychiatr Scand. 2007;115(6):451–7.

61. Wigman JTW, van Os J, Thiery E, Derom C, Collip D, Jacobs N, et al. Psychiatric Diagnosis Revisited: Towards a System of Staging and Profiling Combining Nomothetic and Idiographic Parameters of Momentary Mental States. PLOS ONE. 2013 Mar 28;8(3):e59559.

62. Barge-Schaapveld D, Nicholson NA. Effects of antidepressant treatment on the quality of daily life: an experience sampling study. J Clin Psychiatry. 2002;63:477–85.

63. Barge-Schaapveld DQ, Nicolson NA, van der Hoop RG, DeVries MW. Changes in daily life experience associated with clinical improvement in depression. J Affect Disord. 1995;34(2):139–54.

64. Depp CA, Moore RC, Dev SI, Mausbach BT, Eyler LT, Granholm EL. The temporal course and clinical correlates of subjective impulsivity in bipolar disorder as revealed through ecological momentary assessment. J Affect Disord. 2016;193:145–50.

65. Havermans R, Nicolson NA, Berkhof J, deVries MW. Mood reactivity to daily events in patients with remitted bipolar disorder. Psychiatry Res. 2010 Aug;179(1):47–52.

66. Höhn P, Menne-Lothmann C, Peeters F, Nicolson NA, Jacobs N, Derom C, et al. Moment-to-Moment Transfer of Positive Emotions in Daily Life Predicts Future Course of Depression in Both General Population and Patient Samples. Markou A, editor. PLoS ONE. 2013 Sep 23;8(9):e75655.

67. Palmier-Claus JE, Dunn G, Taylor H, Morrison AP, Lewis SW. Cognitive-self consciousness and metacognitive beliefs: Stress sensitization in individuals at ultra-high risk of developing psychosis: *Cognitive-self consciousness and metacognitive beliefs*. Br J Clin Psychol. 2013 Mar;52(1):26–41.

68. Palmier-Claus JE, Taylor PJ, Gooding P, Dunn G, Lewis SW. Affective variability predicts suicidal ideation in individuals at ultra-high risk of developing psychosis: An experience sampling study: Affective variability as a predictor of suicidal ideation. Br J Clin Psychol. 2012 Mar;51(1):72–83.

69. Pavlickova H, Varese F, Smith A, Myin-Germeys I, Turnbull OH, Emsley R, et al. The Dynamics of Mood and Coping in Bipolar Disorder: Longitudinal Investigations of the Inter-Relationship between Affect, Self-Esteem and Response Styles. Zhang XY, editor. PLoS ONE. 2013 Apr 26;8(4):e62514.

70. Peeters F, Berkhof J, Delespaul P, Rottenberg J, Nicolson NA. Diurnal mood variation in major depressive disorder. Emotion. 2006;6(3):383–91.

71. Peeters F, Berkhof J, Rottenberg J, Nicolson NA. Ambulatory emotional reactivity to negative daily life events predicts remission from major depressive disorder. Behav Res Ther. 2010 Aug;48(8):754–60.

72. Ainsworth J, Palmier-Claus JE, Machin M, Barrowclough C, Dunn G, Rogers A, et al. A Comparison of Two Delivery Modalities of a Mobile Phone-Based Assessment for Serious Mental Illness: Native Smartphone Application vs Text-Messaging Only Implementations. J Med Internet Res. 2013 Apr 5;15(4):e60.

73. Aldinger M, Stopsack M, Ulrich I, Appel K, Reinelt E, Wolff S, et al. Neuroticism developmental courses-implications for depression, anxiety and everyday emotional experience; a prospective study from adolescence to young adulthood. BMC Psychiatry. 2014;14(1):210.

74. Bak M, Drukker M, van Os J, Delespaul P, Myin-Germeys I. Daily life moment-to-moment variation in coping in people with a diagnosis of schizophrenia: state within trait psychosis. Psychosis. 2012;4(2):115–25.

75. Barge-Schaapveld DQ, Nicholson NA, Berkhof J. Quality of life in depression: daily life determinants and variability. Psychiatry Res. 1999;88(3):173–9.

76. Barrantes-Vidal N, Chun CA, Myin-Germeys I, Kwapil TR. Psychometric schizotypy predicts psychotic-like, paranoid, and negative symptoms in daily life. J Abnorm Psychol. 2013;122(4):1077–87.

77. Bentall RP, Myin-Germeys I, Smith A, Knowles R, Jones SH, Smith T, et al. Hypomanic Personality, Stability of Self-Esteem and Response Styles to Negative Mood: Hypomania, Self-Esteem and Response Styles to Negative Mood. Clin Psychol Psychother. 2011 Sep;18(5):397–410.

78. Ben-Zeev D, Young MA. Accuracy of Hospitalized Depressed Patients’ and Healthy Controls’ Retrospective Symptom Reports: An Experience Sampling Study. J Nerv Ment Dis [Internet]. 2010;198(4). Available from: http://journals.lww.com/jonmd/Fulltext/2010/04000/Accuracy_of_Hospitalized_Depressed_Patients__and.6.aspx

79. Ben-Zeev D, Ellington K, Swendsen J, Granholm E. Examining a Cognitive Model of Persecutory Ideation in the Daily Life of People With Schizophrenia: A Computerized Experience Sampling Study. Schizophr Bull. 2011 Nov 1;37(6):1248–56.

80. Ben-Zeev D, McHugo GJ, Xie H, Dobbins K, Young MA. Comparing Retrospective Reports to Real-Time/Real-Place Mobile Assessments in Individuals With Schizophrenia and a Nonclinical Comparison Group. Schizophr Bull. 2012 May 1;38(3):396–404.

81. Blood EA, Shrier LA. The temporal relationship between momentary affective states and condom use in depressed adolescents. Arch Sex Behav. 2013;42(7):1209–16.

82. Blum LH, Vakhrusheva J, Saperstein A, Khan S, Chang RW, Hansen MC, et al. Depressed mood in individuals with schizophrenia: A comparison of retrospective and real-time measures. Psychiatry Res. 2015 Jun;227(2–3):318–23.

83. Brodbeck J, Bachmann MS, Brown A, Znoj HJ. Effects of depressive symptoms on antecedents of lapses during a smoking cessation attempt: an ecological momentary assessment study. Addiction. 2014;109(8):1363–70.

84. Brown LH, Strauman T, Barrantes-Vidal N, Silvia PJ, Kwapil TR. An Experience-Sampling Study of Depressive Symptoms and Their Social Context: J Nerv Ment Dis. 2011 Jun;199(6):403–9.

85. Clasen PC, Fisher AJ, Beevers CG. Mood-Reactive Self-Esteem and Depression Vulnerability: Person-Specific Symptom Dynamics via Smart Phone Assessment. PloS One. 2015;10(7):e0129774.

86. Demiralp E, Thompson RJ, Mata J, Jaeggi SM, Buschkuehl M, Barrett LF, et al. Feeling Blue or Turquoise? Emotional Differentiation in Major Depressive Disorder. Psychol Sci. 2012;23(11):1410–6.

87. Depp CA, Ceglowski J, Wang VC, Yaghouti F, Mausbach BT, Thompson WK, et al. Augmenting psychoeducation with a mobile intervention for bipolar disorder: A randomized controlled trial. J Affect Disord. 2015 Mar;174:23–30.

88. deVries MW, Delespaul PA. Time, context, and subjective experiences in schizophrenia. Schizophr Bull. 1989;15(2):233–44.

89. e Sa DV, Wearden A, Hartley S, Emsley R, Barrowclough C. Expressed Emotion and behaviourally controlling interactions in the daily life of dyads experiencing psychosis. Psychiatry Res. 2016;245:406–13.

90. Gard DE, Sanchez AH, Cooper K, Fisher M, Garrett C, Vinogradov S. Do people with schizophrenia have difficulty anticipating pleasure, engaging in effortful behavior, or both? J Abnorm Psychol. 2014;123(4):771–82.

91. Geschwind N, Nicolson NA, Peeters F, van Os J, Barge-Schaapveld D, Wichers M. Early improvement in positive rather than negative emotion predicts remission from depression after pharmacotherapy. Eur Neuropsychopharmacol. 2011 Mar;21(3):241–7.

92. Granholm E, Ben-Zeev D, Fulford D, Swendsen J. Ecological Momentary Assessment of social functioning in schizophrenia: Impact of performance appraisals and affect on social interactions. Schizophr Res. 2013 Apr;145(1–3):120–4.

93. Gruber J, Kogan A, Mennin D, Murray G. Real-world emotion? An experience-sampling approach to emotion experience and regulation in bipolar I disorder. J Abnorm Psychol. 2013;122(4):971.

94. Hartley S, Haddock G, Vasconcelos e Sa D, Emsley R, Barrowclough C. The influence of thought control on the experience of persecutory delusions and auditory hallucinations in daily life. Behav Res Ther. 2015 Feb;65:1–4.

95. Havermans R, Nicolson NA. Daily hassles, uplifts, and time use in individuals with bipolar disorder in remission. J Nerv Ment Dis. 2007;195(9):745–51.

96. Hernaus D, Collip D, Lataster J, Viechtbauer W, Myin E, Ceccarini J, et al. Psychotic reactivity to daily life stress and the dopamine system: A study combining experience sampling and [^18^F]fallypride positron emission tomography. J Abnorm Psychol. 2015;124(1):27–37.

97. Huffziger S, Ebner-Priemer U, Eisenbach C, Koudela S, Reinhard I, Zamoscik V, et al. Induced ruminative and mindful attention in everyday life: An experimental ambulatory assessment study. J Behav Ther Exp Psychiatry. 2013;44(3):322–8.

98. Hung S, Li M-S, Chen Y-L, Chiang J-H, Chen Y-Y, Hung GC-L. Smartphone-based ecological momentary assessment for Chinese patients with depression: An exploratory study in Taiwan. Asian J Psychiatry. 2016;23:131–6.

99. Husky MM, Gindre C, Mazure CM, Brebant C, Nolen-Hoeksema S, Sanacora G, et al. Computerized ambulatory monitoring in mood disorders: Feasibility, compliance, and reactivity. Psychiatry Res. 2010 Jul;178(2):440–2.

100. Husky MM, Grondin OS, Swendsen JD. The relation between social behavior and negative affect in psychosis-prone individuals: an experience sampling investigation. Eur Psychiatry. 2004;19(1):1–7.

101. Husky MM, Mazure CM, Maciejewski PK, Swendsen JD. A daily life comparison of sociotropy-autonomy and hopelessness theories of depression. Cogn Ther Res. 2007;31(5):659–76.

102. Husky M, Olié E, Guillaume S, Genty C, Swendsen J, Courtet P. Feasibility and validity of ecological momentary assessment in the investigation of suicide risk. Psychiatry Res. 2014 Dec;220(1–2):564–70.

103. Jahng S, Wood PK, Trull TJ. Analysis of affective instability in ecological momentary assessment: Indices using successive difference and group comparison via multilevel modeling. Psychol Methods. 2008;13(4):354–75.

104. Johnson SL, Cuellar AK, Ruggero C, Winett-Perlman C, Goodnick P, White R, et al. Life events as predictors of mania and depression in bipolar I disorder. J Abnorm Psychol. 2008 May;117(2):268–77.

105. Kendall AD, Wilt J, Walls CE, Scherer EA, Beardslee WR, Revelle W, et al. The Social Context of Positive and Negative Affective States in Depressed Youth. J Soc Clin Psychol. 2014 Nov;33(9):805–30.

106. Kimhy D, Vakhrusheva J, Khan S, Chang RW, Hansen MC, Ballon JS, et al. Emotional granularity and social functioning in individuals with schizophrenia: an experience sampling study. J Psychiatr Res. 2014;53:141–8.

107. Kircanski K, Thompson RJ, Sorenson J, Sherdell L, Gotlib IH. The everyday dynamics of rumination and worry: precipitant events and affective consequences. Cogn Emot. 2017 Jan 20;1–13.

108. Köhling J, Moessner M, Ehrenthal JC, Bauer S, Cierpka M, Kämmerer A, et al. Affective Instability and Reactivity in Depressed Patients With and Without Borderline Pathology. J Personal Disord. 2016 Dec;30(6):776–95.

109. Koval P, Brose A, Pe ML, Houben M, Erbas Y, Champagne D, et al. Emotional inertia and external events: The roles of exposure, reactivity, and recovery. Emotion. 2015;15(5):625.

110. Kuepper R, Oorschot M, Myin‐Germeys I, Smits M, van Os J, Henquet C. Is psychotic disorder associated with increased levels of craving for cannabis? An Experience Sampling study. Acta Psychiatr Scand. 2013;128(6):448–56.

111. Kuppens P, Oravecz Z, Tuerlinckx F. Feelings change: Accounting for individual differences in the temporal dynamics of affect. J Pers Soc Psychol. 2010;99(6):1042–60.

112. Kwapil TR, Brown LH, Silvia PJ, Myin-Germeys I, Barrantes-Vidal N. The expression of positive and negative schizotypy in daily life: an experience sampling study. Psychol Med. 2012 Dec;42(12):2555–66.

113. Lardinois M, Myin‐Germeys I, Bak M, Mengelers R, Van Os J, Delespaul PA. The dynamics of symptomatic and non‐symptomatic coping with psychotic symptoms in the flow of daily life. Acta Psychiatr Scand. 2007;116(1):71–5.

114. Lee-Flynn SC, Pomaki G, DeLongis A, Biesanz JC, Puterman E. Daily Cognitive Appraisals, Daily Affect, and Long-Term Depressive Symptoms: The Role of Self-Esteem and Self-Concept Clarity in the Stress Process. Pers Soc Psychol Bull. 2011 Feb;37(2):255–68.

115. Lüdtke T, Kriston L, Schröder J, Lincoln TM, Moritz S. Negative affect and a fluctuating jumping to conclusions bias predict subsequent paranoia in daily life: An online experience sampling study. J Behav Ther Exp Psychiatry [Internet]. 2016 Aug [cited 2017 Apr 7]; Available from: http://linkinghub.elsevier.com/retrieve/pii/S0005791616301021

116. Mata J, Thompson RJ, Jaeggi SM, Buschkuehl M, Jonides J, Gotlib IH. Walk on the bright side: Physical activity and affect in major depressive disorder. J Abnorm Psychol. 2012;121(2):297–308.

117. McCormick BP, Snethen G, Lysaker PH. Emotional episodes in the everyday lives of people with schizophrenia: The role of intrinsic motivation and negative symptoms. Schizophr Res. 2012 Dec;142(1–3):46–51.

118. Moberly NJ, Watkins ER. Ruminative self-focus and negative affect: an experience sampling study. J Abnorm Psychol. 2008;117(2):314.

119. Moran EK, Culbreth AJ, Barch DM. Ecological momentary assessment of negative symptoms in schizophrenia: Relationships to effort-based decision making and reinforcement learning. J Abnorm Psychol. 2017;126(1):96–105.

120. Myin-Germeys I, Delespaul P, Van Os J. Behavioural sensitization to daily life stress in psychosis. Psychol Med. 2005 May;35(5):733–41.

121. Olino TM, McMakin DL, Morgan JK, Silk JS, Birmaher B, Axelson DA, et al. Reduced reward anticipation in youth at high-risk for unipolar depression: A preliminary study. Dev Cogn Neurosci. 2014 Apr;8:55–64.

122. Oorschot M, Lataster T, Thewissen V, Lardinois M, van Os J, Delespaul PAEG, et al. Symptomatic remission in psychosis and real-life functioning. Br J Psychiatry. 2012 Sep 1;201(3):215–20.

123. Palmier-Claus JE, Dunn G, Lewis SW. Emotional and symptomatic reactivity to stress in individuals at ultra-high risk of developing psychosis. Psychol Med. 2012 May;42(05):1003–12.

124. Peeters F, Nicholson NA, Berkhof J. Cortisol Responses to Daily Events in Major Depressive Disorder: Psychosom Med. 2003 Sep;65(5):836–41.

125. Peters E, Lataster T, Greenwood K, Kuipers E, Scott J, Williams S, et al. Appraisals, psychotic symptoms and affect in daily life. Psychol Med. 2012 May;42(05):1013–23.

126. Reininghaus U, Kempton MJ, Valmaggia L, Craig TKJ, Garety P, Onyejiaka A, et al. Stress Sensitivity, Aberrant Salience, and Threat Anticipation in Early Psychosis: An Experience Sampling Study. Schizophr Bull. 2016 May;42(3):712–22.

127. van Roekel E, Bennik EC, Bastiaansen JA, Verhagen M, Ormel J, Engels RCME, et al. Depressive Symptoms and the Experience of Pleasure in Daily Life: An Exploration of Associations in Early and Late Adolescence. J Abnorm Child Psychol. 2016 Jul;44(5):999–1009.

128. Sagar KA, Dahlgren MK, Racine MT, Dreman MW, Olson DP, Gruber SA. Joint Effects: A Pilot Investigation of the Impact of Bipolar Disorder and Marijuana Use on Cognitive Function and Mood. Hashimoto K, editor. PLOS ONE. 2016 Jun 8;11(6):e0157060.

129. Schwartz S, Schultz S, Reider A, Saunders EFH. Daily mood monitoring of symptoms using smartphones in bipolar disorder: A pilot study assessing the feasibility of ecological momentary assessment. J Affect Disord. 2016 Feb;191:88–93.

130. Sitko K, Varese F, Sellwood W, Hammond A, Bentall R. The dynamics of attachment insecurity and paranoid thoughts: An experience sampling study. Psychiatry Res. 2016 Dec;246:32–8.

131. Snippe E, Simons CJP, Hartmann JA, Menne-Lothmann C, Kramer I, Booij SH, et al. Change in daily life behaviors and depression: Within-person and between-person associations. Health Psychol. 2016;35(5):433–41.

132. So SH, Peters ER, Swendsen J, Garety PA, Kapur S. Detecting improvements in acute psychotic symptoms using experience sampling methodology. Psychiatry Res. 2013 Nov;210(1):82–8.

133. Solhan MB, Trull TJ, Jahng S, Wood PK. Clinical assessment of affective instability: Comparing EMA indices, questionnaire reports, and retrospective recall. Psychol Assess. 2009;21(3):425–36.

134. Swendsen JD. Anxiety, depression, and their comorbidity: An experience sampling test of the helplessness-hopelessness theory. Cogn Ther Res. 1997;21(1):97–114.

135. Swendsen JD. The helplessness–hopelessness theory and daily mood experience: An idiographic and cross-situational perspective. J Pers Soc Psychol. 1998;74(5):1398.

136. Swendsen J, Ben-Zeev D, Granholm E. Real-time electronic ambulatory monitoring of substance use and symptom expression in schizophrenia. Am J Psychiatry. 2011;168(2):202–209.

137. Takano K, Tanno Y. Diurnal variation in rumination. Emotion. 2011;11(5):1046–58.

138. Talbot LS, Stone S, Gruber J, Hairston IS, Eidelman P, Harvey AG. A test of the bidirectional association between sleep and mood in bipolar disorder and insomnia. J Abnorm Psychol. 2012;121(1):39–50.

139. Thewissen V, Bentall RP, Lecomte T, van Os J, Myin-Germeys I. Fluctuations in self-esteem and paranoia in the context of daily life. J Abnorm Psychol. 2008;117(1):143–53.

140. Thompson RJ, Mata J, Jaeggi SM, Buschkuehl M, Jonides J, Gotlib IH. The everyday emotional experience of adults with major depressive disorder: Examining emotional instability, inertia, and reactivity. J Abnorm Psychol. 2012;121(4):819–29.

141. Udachina A, Varese F, Oorschot M, Myin-Germeys I, Bentall RP. Dynamics of self-esteem in “poor-me” and “bad-me” paranoia. J Nerv Ment Dis. 2012;200(9):777–783.

142. Vachon H, Bourbousson M, Deschamps T, Doron J, Bulteau S, Sauvaget A, et al. Repeated self-evaluations may involve familiarization: An exploratory study related to Ecological Momentary Assessment designs in patients with major depressive disorder. Psychiatry Res. 2016 Nov;245:99–104.

143. Verdoux H, Gindre C, Sorbara F, Tournier M, Swendsen JD. Effects of cannabis and psychosis vulnerability in daily life: an experience sampling test study. Psychol Med. 2003;33(01):23–32.

144. Vranceanu A-M, Gallo LC, Bogart LM. Depressive symptoms and momentary affect: the role of social interaction variables. Depress Anxiety. 2009 May;26(5):464–70.

145. Wenze SJ, Gunthert KC, Ahrens AH, Bos TT. Biases in Short-Term Mood Prediction in Individuals with Depression and Anxiety Symptoms. Individ Differ Res IDR. 2013;11(3):91.

146. White ME, Shih JH. A Daily Diary Study of Co-Rumination, Stressful Life Events, and Depressed Mood in Late Adolescents. J Clin Child Adolesc Psychol. 2012 Sep;41(5):598–610.

147. Wichers M, Peeters F, Geschwind N, Jacobs N, Simons CJP, Derom C, et al. Unveiling patterns of affective responses in daily life may improve outcome prediction in depression: A momentary assessment study. J Affect Disord. 2010 Jul;124(1–2):191–5.

148. Wichers M, Peeters F, Rutten BPF, Jacobs N, Derom C, Thiery E, et al. A time-lagged momentary assessment study on daily life physical activity and affect. Health Psychol. 2012;31(2):135–44.

149. Ramsey AT, Wetherell JL, Depp C, Dixon D, Lenze E. Feasibility and Acceptability of Smartphone Assessment in Older Adults with Cognitive and Emotional Difficulties. J Technol Hum Serv. 2016 Apr 2;34(2):209–23.

150. Nakagawa S, Poulin R, Mengersen K, Reinhold K, Engqvist L, Lagisz M, et al. Meta‐analysis of variation: ecological and evolutionary applications and beyond. Methods Ecol Evol. 2015;6(2):143–52.
